# Evaluation of silvestrol as a potential therapeutic agent for pediatric COVID-19: an interpreted computational and phytochemistry approach

**DOI:** 10.3389/fphar.2025.1673591

**Published:** 2025-09-17

**Authors:** Yi Zhang, Shanshan Pu, Hui Wang

**Affiliations:** Department of Pediatrics, Pu’er People’s Hospital, Pu’er, China

**Keywords:** COVID-19, silvestrol, molecular docking, admet, MD simulation, DFT

## Abstract

**Background:**

The persistent COVID-19 disease, induced by SARS-CoV-2, sparked great questions about the safety and efficacy of the existing therapies in pediatric patients. The currently available antiviral drugs for treating COVID-19, either remdesivir or monoclonal antibodies, are primarily designed for adults. In many cases, their development has been hindered by concerns about safety and pediatric populations.

**Objectives:**

In the present study, we consider Silvestrol, a natural product derived from *Euphorbia hirta*, as a potential treatment for pediatric COVID-19.

**Methods:**

The molecular docking studies revealed that Silvestrol exhibits a highly competitive binding affinity of −7.5 kcal/mol with the receptor-binding domain (RBD) of the SARS-CoV-2 spike protein, indicating that it may inhibit viral entry. In order to learn more about the dynamics of this interaction, the molecular dynamics (MD) simulations were carried out, which proved that the protein was stabilized in 150 ns, whereas the ligand showed conformational changes to be fit in the binding pocket, and finally stabilizing. The characterization of the pharmacophore also revealed important interaction points, including four hydrogen bond donors, 12 hydrogen bond acceptors, and eight hydrophobic sites, which increase its binding potential.

**Results:**

The favorable ADMET analysis predicted the pharmacokinetic properties of Silvestrol, which exhibited tumor-killing characteristics *in vitro* and *in vivo* activities, and an LD50 of 2,300 mg/kg (toxicity 5), implying a high safety margin. Most toxicity endpoints of Silvestrol were likely to be inactive; however, there was a chance of immunotoxicity and nutritional toxicity, which require further investigation. Its reactivity in antiviral interactions has been confirmed by its reasonable value of 0.20606 eV obtained through the DFT analysis.

**Conclusion:**

The observations suggest that Silvestrol is a promising agent for treating COVID-19 in children, as it exhibits a potent antiviral effect, low toxicity, and favorable pharmacokinetics. Further preclinical and clinical testing is needed to demonstrate its effectiveness and safety in children.

## Introduction

The introduction of SARS-CoV-2 has had significant consequences on global health, with children, although the cases are generally milder in comparison to adults, remaining under significant threat. Pediatric cases of COVID-19 exhibit variable symptomatology, characterized by fever, cough, sore throat, fatigue, and abdominal symptoms such as diarrhea and vomiting (Rasmussen and Popescu, 2021). A substantial share of children can also experience severe complications, such as Multisystem Inflammatory Syndrome in Children (MIS-C), which is characterized by inflammation of multiple organs, including the cardiovascular system, respiratory system, lungs, kidneys, and brain (Molloy et al., 2023). Although children experience fewer cases of severe disease than adults, the possibility of severe cases in children, especially in the context of new variants, renders the issue of practical and specific therapeutics for children a priority (Bakker et al., 2021).

Current cures for COVID-19 are mainly available to adults, and few options exist for children. Adults received approved antiviral agents like remdesivir, which in children have shown to be of safety concerns because of their side effects, which include hepatotoxicity and renal dysfunction (Beigel et al., 2020). Monoclonal antibodies, too, have been found to have potential in lowering the viral load and yielding better results; however, they also have their problems, such as complications related to dosing, as well as allergies in children (Castagnoli et al., 2020). With these constraints, safer and more effective antiviral drugs have to be specially customized and directed towards pediatric applications.

In humans, infection begins with the entry of SARS-CoV-2 into the cell through the spike protein, which interacts with the angiotensin-converting enzyme 2 (ACE2) receptor on the host cell membrane (Verdecchia et al., 2020). Significantly contributing to the process of viral entry, the receptor-binding domain (RBD) is located on the spike protein and is considered a strong candidate for the development of antiviral therapeutics (Tai et al., 2020). Interfering with RBD-ACE2 interaction by inhibiting the RBD would disrupt the viral entry process and stop the infection. Focused on this essential protein, it is possible to minimize the viral burden and suppress the effects of the pathology, particularly in endangered pediatric cohorts, where high-risk conventional approaches become exceptionally dangerous (Van den Eynde, 2020).

Medicinal plants represent the ancient foundation of folk medicine, providing a substantial supply of bioactive substances with antiviral activity (Ben-Shabat et al., 2020). Phytochemicals such as flavonoids, alkaloids, and terpenoids have been shown to inhibit viral growth and alter immune processes, making them invaluable in the quest to discover new therapeutic agents (Ben-Shabat et al., 2020). Their application is also important, especially in the field of pediatrics, where safety is of paramount importance in the use of chemical drugs. Plants such as *Euphorbia hirta*, which produce Silvestrol, also contain substances that have shown antiviral effectiveness without causing the serious side effects typical of most synthetic pharmaceuticals (Biedenkopf et al., 2017). Silvestrol is a natural flavonoid that exhibits potent antiviral activity against SARS-CoV-2, primarily due to its ability to inhibit the eukaryotic translation initiation factor eIF4A, which is critical for the production of viral proteins. It was demonstrated in preclinical studies that Silvestrol has a favorable toxicity profile, and the presence of minimal side effects indicates that it could be a promising candidate for the treatment of children (Müller et al., 2018).

Silvestrol is a flavagline natural product featuring a distinctive cyclopenta [b] benzofuran core and a rare dioxanyl ether side chain, originally isolated from *Aglaia foveolata* and related *Aglaia* species (Saradhi et al., 2011). It demonstrates remarkable potency as an inhibitor of the RNA helicase eIF4A, effectively stalling cap-dependent mRNA translation, making it highly active in both anticancer and antiviral studies (Patton et al., 2014). For example, silvestrol induces autophagy and caspase-dependent apoptosis in melanoma cells by disrupting critical cell-cycle regulators, and suppresses oncogenic pathways in hematologic malignancies with limited toxicity to normal immune cells (Chen et al., 2016). Despite its pronounced biological activity, silvestrol exhibits markedly poor oral bioavailability (∼1.7%) in murine models, largely due to rapid metabolic degradation and efflux transporter activity; conversely, intraperitoneal delivery achieves full systemic availability, highlighting key pharmacokinetic constraints (Saradhi et al., 2011). *In vitro* assessments further reveal that while silvestrol has low membrane permeability, it maintains good stability in liver microsomes and demonstrates efficient cellular accumulation particularly in cell lines with low P-glycoprotein expression—indicating favorable intracellular retention despite permeability challenges (Schiffmann et al., 2022). Such structural, mechanistic, and pharmacokinetic considerations provide crucial justification for our focused computational analyses, as they directly inform modeling of silvestrol’s binding interactions, pharmacodynamics, and ADMET properties.

The objectives of the present investigation are to characterize Silvestrol as a potential therapy for pediatric COVID-19 by quantifying its binding to the RBD of the SARS-CoV-2 spike protein and by examining its safety profile through ADMET (Absorption, Distribution, Metabolism, Excretion, and Toxicity) evaluation. Our question of interest is to determine whether Silvestrol will exhibit practical antiviral efficacy with apoptosis-inducing properties and a safety profile acceptable in a pediatric setting. By allowing us to gain an understanding of the molecular responses and assess the safety and efficacy of Silvestrol, the study has the potential to result in a more secure option for treating COVID-19 in children and possibly other viral diseases as well.

### Methodology

#### Retrieval of target protein structure

The three-dimensional crystal structure of the receptor-binding domain (RBD) from the SARS-CoV-2 spike protein was retrieved from Protein Data Bank under accession 7DQA (https://www.rcsb.org/structure/7DQA) (Velankar et al., 2021; Hussain et al., 2024). The RBD coordinate file was saved in PDB format and loaded into docking software for subsequent ligand-interaction studies. Because the data were obtained directly from the experiment, no homology modeling or energy minimization was necessary.

### Validation of protein 3D structure

To assess the geometry and reliability of the 3D model of the SARS-CoV-2 spike protein (PDB ID 7DQA), we examined it with a Ramachandran plot and an ERRAT test, both of which are accessible through the SAVES web server (https://saves.mbi.ucla.edu/) (Asrar et al., 2025; Colovos et al., 1993). The Ramachandran plot, by mapping backbone dihedral angles, allows researchers to quickly assess whether most residues occupy energetically favored conformational regions, a key indicator of local structural stability. In a complementary role, the ERRAT algorithm plays a crucial part in the process, meticulously analyzing patterns of non-bonded interactions across the entire structure. It flags regions where atom-pair scores deviate from expected statistical norms, thereby identifying potential issues. The convergence of results from these distinct assessments provides robust evidence for the model’s accuracy, establishing it as a dependable platform for subsequent molecular simulations and design experiments.

### Prediction of the active site of targeted protein

Using PrankWeb, a publicly accessible web tool (https://prankweb.cz), we mapped the binding pockets and active sites on the SARS-CoV-2 spike-protein receptor-binding domain (PDB ID 7DQA) (Jakubec et al., 2022). This algorithm highlighted peptides scattered across the protein surface that are energetically favorable for ligand attachment, thereby guiding subsequent drug-development efforts. These predicted pcokets, with their potential to mediate the strongest contacts with candidate antiviral molecules, offer hope for the development of particular therapeutic hits. Alongside the residue identification, PrankWeb_RCR provided grid-box parameters for docking, ensuring that the receptor dimensions during virtual screening.

### Retrieval of phytochemical 3D structures

Viruses cause severe human disease and strain health systems globally. To identify the most promising antiviral compounds, we curated literature to select phytochemicals with antiviral activity and no safety concerns. These compounds, which originated primarily from edible plants, traditional remedies, or synthetic versions to ease supply, were subjected to a thorough screening process. Motivated by observed activity against the spike protein of SARS-CoV-2, investigators obtained uniform 3D coordinates for each molecule from the PubChem repository (https://pubchem.ncbi.nlm.nih.gov/) (Asrar et al., 2025; Kim et al., 2016). PubChem offers multidimensional structural files derived from X-ray crystallography and nuclear magnetic resonance (NMR), which capture plausible molecular conformations under physiological conditions.

### Molecular dynamics docking of antiviral phytochemicals and molecular interactions analysis

The 10 compounds were docked to the receptor-binding domain of the SARS-CoV-2 spike protein (PDB ID: 7DQA) by AutoDock Vina, with bindings checked in MOE for cross-validation of scoring functions (Huey et al., 2012; Vilar et al., 2008; Naveed et al., 2024a). The protocol began with ligand semi-flexible preparation, conversion to the PDBQT format. AutoDock Vina estimates the strength with which each ligand associates with the target receptor, offering a way to predict the most plausible docking orientations based solely on computed binding energy values. In the present investigation, the search region was defined by a rectangular box measuring 78.0483 78.3086 and 77.9837 Ǻ along the X, Y, and Z-axes, respectively, a volume that faithfully encloses the receptor’s active site. These settings were chosen to maximize coverage of the surface pocket and therefore to minimize the risk of overlooking potentially relevant binding modes.

Complementing the AutoDock workflow, docking analyses were also carried out in the MOE environment, an integrated platform that combines several tools for advanced molecular modeling. Before calculations, each ligand was energy-minimized to relieve any steric strain, after which receptor-ligand complexes were examined using MOE’s tiered scoring protocol, which weighs geometry, sterics, and van der Waals interactions. This approach simultaneously explores multiple ligand conformations and allows partial flexibility of the receptor, thereby producing robust estimates of the favored binding modes for the selected antiviral phytochemicals.

Finally, to cross-check the docking predictions and to visualize the molecular contacts, output files were processed with LigPlot PyMOL and Discovery Studio, which yielded both 2D interaction fingerprints and 3D structural snapshots (Laskowski and Swindells, 2011; Jejurikar and Rohane, 2021; Yuan et al., 2017; Naveed et al., 2024b). These illustrations reveal hydrogen bonds, salt bridges, and hydrophobic patches in detail, showing bond angles and indicating precisely which amino-acid side chains participate in each contact. Such visual information consolidates the energetic models and provides a more holistic view of how the lead compounds engage the receptor interface, thus bolstering confidence in the virtual screening campaign.

### ADMET screening of phytochemicals

The 10 phytochemicals were screened by systematic examination of ADMET (Absorption, Distribution, Metabolism, Excretion, and Toxicity) is critical for predicting how a candidate will move through the body and whether unintended harm may occur. To this end, the ADMET characteristics of the lead antiviral plant extracts were modeled using the open-access tools SwissADME (http://www.swissadme.ch/), and ProTox 3.0, (https://tox.charite.de/protox3/index.php?site=compound_input) (Daina et al., 2017; Banerjee et al., 2024). Qualitative estimates for oral bioavailability, blood-brain-barrier permeation, inhibition or induction of cytochrome P450 isoforms, probable organ-specific toxicity, and overall risk-grade scores. Combining these insights, researchers prioritized candidates with acceptable pharmacokinetic profiles and an acceptable safety margin for progression into laboratory studies.

### Molecular dynamics (MD) simulation and MMGBSA analysis of receptor protein and lead antiviral compound

Molecular dynamics (MD) simulations, coupled with the MMGBSA energy assessment, were conducted within the Desmond suite, a comprehensive software package provided by Schrödinger, LLC (Ferreira et al., 2015). To establish a baseline binding geometry, the lead antiviral compound was first docked against the receptor-binding domain of the SARS-CoV-2 spike protein (PDB 7DQA); this docking run identified a preferred orientation within the protein’s active pocket and thereby anchored the ligand in the computational system for subsequent dynamic sampling.

Subsequent molecular dynamics simulations ran for 500 nanoseconds and employed classical Newtonian dynamics to mimic the ligand-receptor binding under physiological conditions. Prior to these runs, the ligand-receptor complex was preprocessed with Maestro’s Protein Preparation Wizard, which optimized, minimized, and filled in any missing residues. Using the System Builder tool, the complex was placed in an orthorhombic TIP3P water box maintained at 300 K and 1 atm. An OPLS_2005 force field described atom interactions. Sodium and chloride ions were added to neutralize the system, and a 0.15 mol/L NaCl concentration was included to replicate intracellular salinity. Before production, an equilibration phase relaxed the system, and trajectory frames were saved every 100 picoseconds for later analysis (Shivakumar et al., 2010; Rahman et al., 2025).

The binding free energy between the SARS-CoV-2 spikes protein receptor (PDB ID: 7DQA) and the test antiviral was estimated using the MMGBSA approach implemented in Desmond (Hussain et al., 2024; Zaheer et al., 2023). This framework combines molecular mechanics with a generalized Born description of solvent effects and a surface-area term to yield an end-state free-energy value for the ligand-receptor complex. Total binding free energy was quantified according to Equation 1, which accounts for contributions from the solute internal energy, solvation energies, and entropic components.

$$\mathrm{dGBind}=\mathrm{Gcomplex}–\;\left(\mathrm{Gprotein}+\mathrm{Gligand}\right)$$ (1)

Where dGbind = binding free energy, Gcomplex = free energy of the complex, Gprotein = free energy of the target protein, and Gligand = free energy of the ligand.

### Pharmacophore characterization of lead antiviral phytochemical

Pharmacophore annotations show the essential spatial and electronic features that enable a small molecule to engage a target receptor at the binding pocket. To examine this relationship, we used Pharmit (https://pharmit.csb.pitt.edu/) to dock the lead antiviral plant compound and its pharmacophore sites (Rahman et al., 2025; Koes, 2018).

### Density functional theory (DFT) analysis of lead antiviral phytochemical

Density Functional Theory (DFT) was used to investigate the electronic properties and energetic stability of the selected lead antiviral phytochemicals. All calculations were carried out with Gaussian 09W, and molecular visualization was performed with GaussView 6.0 (Ali et al., 2025). Geometries were first fully optimized at the B3LYP level using the 6-31G (d,p,++) basis set in order to obtain reliable ground-state structures. DFT reliably captures the distribution and energies of molecular orbitals, allowing for meaningful interpretation of the highest occupied molecular orbital (HOMO) and lowest unoccupied molecular orbital (LUMO) levels. The energetic gap ΔE between these two orbitals was evaluated to provide insights into compound reactivity and relative stability. The band gap was computed according to Equation 2:

$$\Delta E=E_{LUMO}\;‐\;E_{HOMO}$$ (2)

where: ΔE_Lumo_ is the energy of the lowest unoccupied molecular orbital, and ΔE_Homo_ is the energy of the highest occupied molecular orbital.

## Results

### Retrieval and validation of target protein 3D structure

**FIGURE 1:**
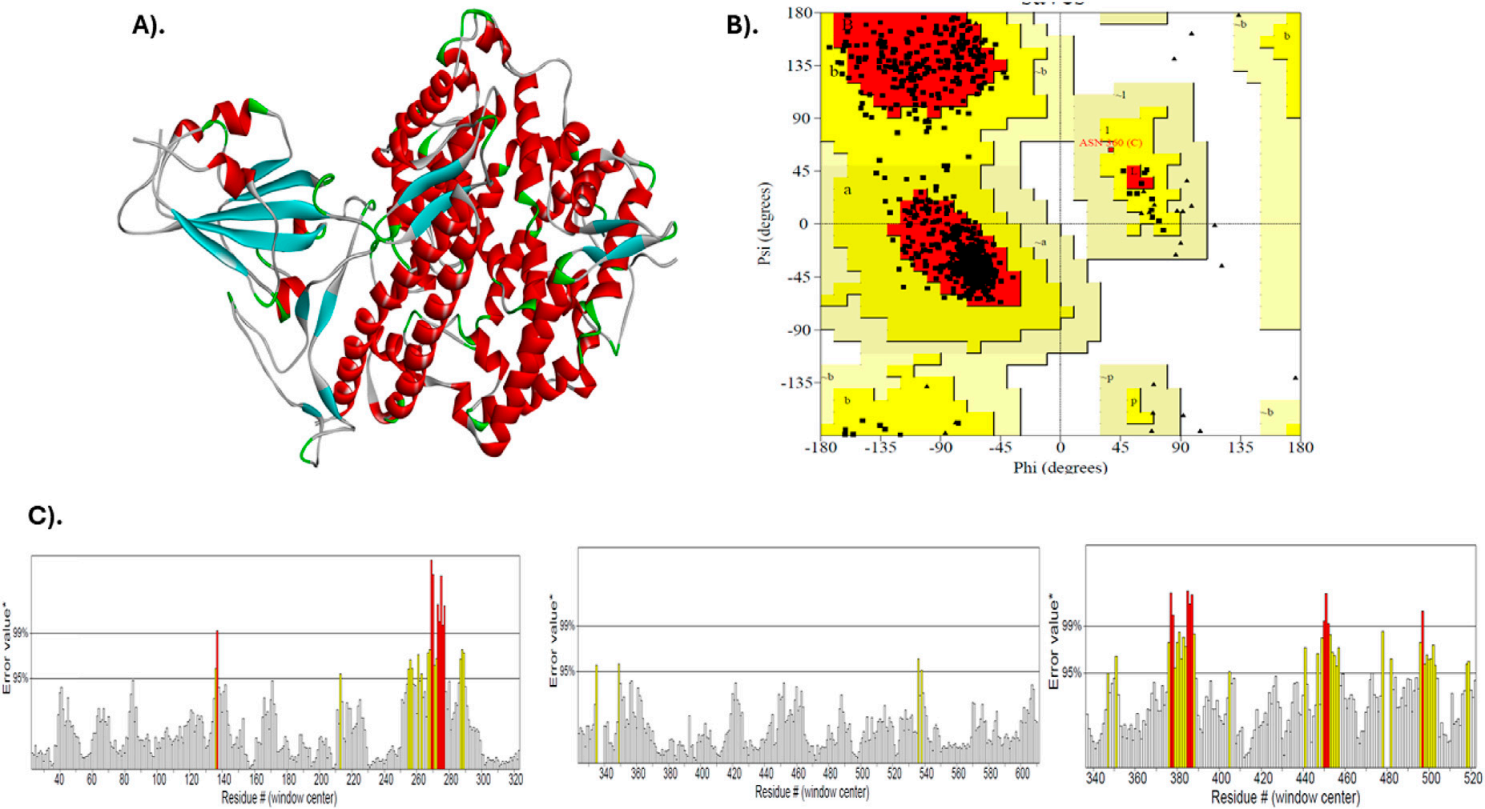
Validation of the 3D structure of SARS-CoV-2 spike protein receptor binding domain (PDB ID: 7DQA). **(A)** The protein’s 3D structure, obtained through electron microscopy with a resolution of 2.80 Å, **(B)** Ramachandran plot showing the distribution of amino acid residues in favored, allowed, and disallowed regions, and **(C)** ERRAT analysis displaying the overall quality factor of the structure (91.525), confirming its high quality for further computational studies.

The structure of the SARS-CoV-2 spike protein receptor binding domain (PDB ID: 7DQA) was downloaded using the Protein Data Bank (Figure 1A). This structure, a viral protein (*homo sapiens*) equivalent to the SARS-CoV-2, was expressed in *Homo sapiens* and resolved at 2.80 Å by electron microscopy. The protein is in a particulate aggregation state, and single-particle techniques were used in its reconstruction. The building contains mutations, and no further modeling was needed. The structure underwent a comprehensive validation process, including the use of the Ramachandran plot and ERRAT (Figures 1B,C). The Ramachandran plot showed that 629 residues (89.2%) were in the most favored regions, 75 residues (10.6%) were in other allowed regions, and only one residue (0.1%) was in the generously allowed regions (Table 1). In prohibited areas, there were no residues. An overall structural validation of the model was achieved by analyzing 705 non-glycine and non-proline residues. ERRAT analysis yielded a general quality factor of 91.525, indicating that the structure is of high quality and suitable for further computational studies.

**TABLE 1:** Ramachandran plot validation results for the 3D structure of the SARS-CoV-2 spike protein receptor binding domain (PDB ID: 7DQA).

Residue type	Number of residues	Percentage (%)
Residues in most favored regions [A, B, L]	629	89.2
Residues in additional allowed regions [a, b, l, p]	75	10.6
Residues in generously allowed regions [∼a, ∼b, ∼l, ∼p]	1	0.1
Residues in disallowed regions	0	0.0
Number of non-glycine and non-proline residues	705	100.0

### Prediction of the active site of targeted protein

**FIGURE 2:**
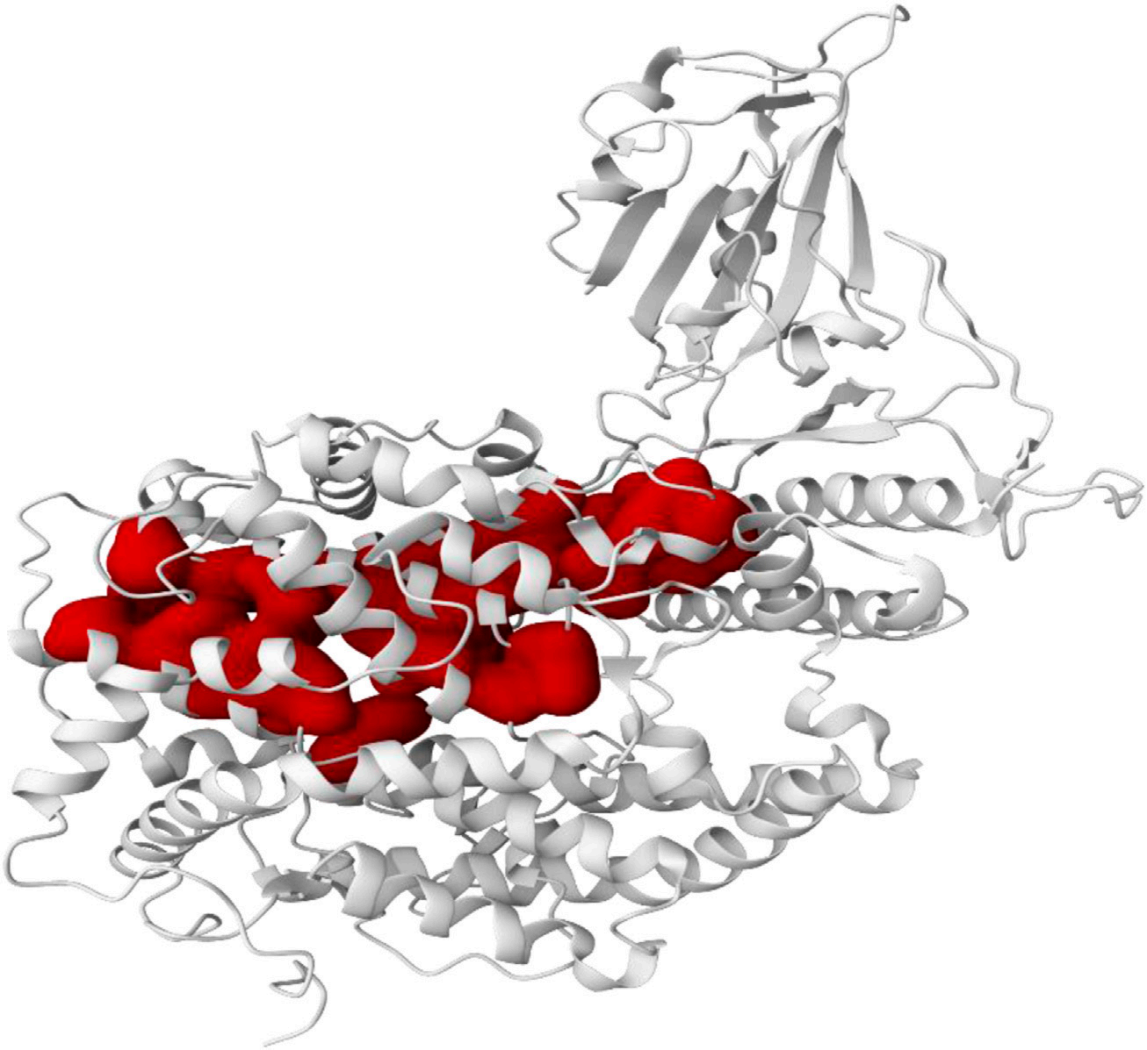
Active site prediction of the SARS-CoV-2 spike protein receptor binding domain (PDB ID: 7DQA). Pocket 1, selected for further study, is highlighted in red to show its optimal binding features and dimensions.

The pocket prediction procedure was then performed to determine the location of the SARS-CoV-2 spike protein receptor binding domain active site (PDB ID: 7DQA) (Figure 2). A total of 14 active pockets were identified, and their respective information is described in the Table 2. Pocket 1, with the largest score of 19.86 and a probability of 0.836, was observed, and analysis continued on it. This pocket had 37 residues and an average conservation of 1.594, with the following dimensions: X = 78.0483, Y = 87.3086, and Z = 77.9837. These lower-scoring and lower-probed pockets were not chosen as the subjects of further studies.

**TABLE 2:** Active site prediction details of the SARS-CoV-2 spike protein receptor binding domain (PDB ID: 7DQA).

No.	Score	Probability	No. of residues	Avg. conservation	Dimensions		
					X	Y	Z
1	19.86	0.836	37	1.594	78.0483	87.3086	77.9837
2	5.18	0.251	17	1.094	88.9552	77.0004	65.8523
3	5.08	0.242	9	2.936	62.9856	83.2438	95.5912
4	3.72	0.148	11	1.793	66.4897	83.5717	84.9133
5	3.57	0.138	10	0.532	81.2554	65.5784	76.0812
6	3.07	0.104	12	1.056	92.1888	65.5608	66.3234
7	1.80	0.033	6	1.896	74.013	85.7305	87.2086
8	1.53	0.022	8	1.355	99.2282	73.5507	40.1127
9	1.49	0.021	8	0.825	79.5758	59.2538	59.6976
10	1.02	0.007	5	2.669	106.7808	71.8253	43.5969
11	1.01	0.007	11	1.883	67.2245	65.2745	101.4841
12	1.00	0.007	11	2.268	60.5961	97.9059	91.078
13	0.70	0.002	6	0.602	85.8429	78.8916	93.8874
14	0.67	0.001	5	1.201	94.959	73.6213	42.879

### Retrieval of phytochemical 3D structures

The PubChem database was used to extract the 10 antiviral phytochemicals selected for participation in the study. These phytochemicals, known for their antiviral properties, were extracted from various natural sources. Such information, which includes the PubChem CID, 3D structures, and natural source(s) for each compound, is provided in Table 3. The selection of these compounds was based on the hope that they would help inhibit the virus. Their 3D structures were downloaded in PDB format for docking and analysis in terms of interactions.

**TABLE 3:** Details of the 10 antiviral phytochemicals retrieved from the PubChem database. The table includes the PubChem CID, 3D structures, and natural source(s) of each compound.

Phytochemical	PubChem CID	3D structure	Natural Source(s)
Castanospermine	54445	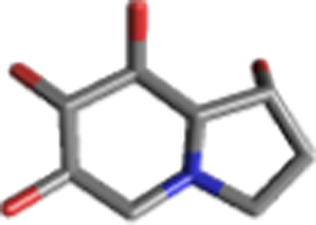	Seeds of *Castanospermum australe* (Moreton Bay chestnut); black beans
Curcumin	969516	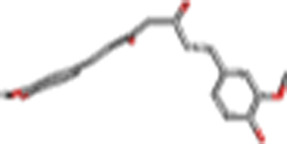	Rhizome of *Curcuma longa* (turmeric)
Emetine	10219	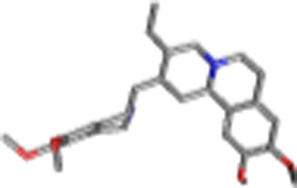	Ipecac root (*Psychotria ipecacuanha*, Rubiaceae)
Emodin-8-glucoside	99649	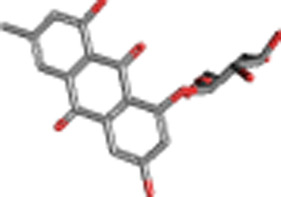	Roots/rhizomes of *Rheum palmatum*, also *Rumex*, *Aloe vera*
Ginkgolic acid	5281858	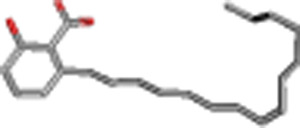	Leaves, seeds, seed coat of *Ginkgo biloba*
Lycorine	72378	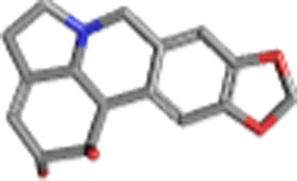	Bulbs/leaves of *Lycoris radiata* and other Amaryllidaceae plants
Moronic acid	489941	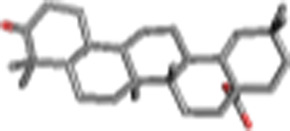	Fruit/seeds of *Morus alba* (white mulberry), *Rhus javanica*, mistletoe (*Phoradendron*)
Patentiflorin A	11785812	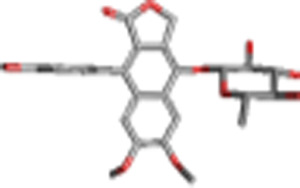	Willow-leaved *Justicia* (e.g., *J. gendarussa*)
Quercetin	5280343	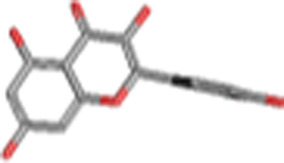	Wide distribution: apples, onions, berries, tea, citrus, kaleetc.
Silvestrol	11787114	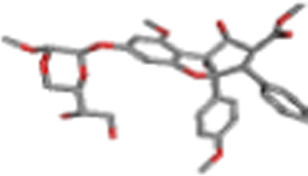	*Aglaia* species (plants in the Meliaceae family) – broad-spectrum sources, *Euphorbia hirta*, natural product

### Molecular dynamics docking of antiviral phytochemicals and molecular interactions analysis

To determine the binding affinities of the identified antiviral phytochemicals against SARS-CoV-2 spike protein receptor binding domain (PDB ID: 7DQA), Molecular dynamics (MD) docking was conducted. The docking of proteins was performed using AutoDock Vina and MOE tools, and their summary results are tabulated in Table 4. Compound Patentiflorin A had the strongest binding affinity of −8.85 kcal/mol, and Emodin-8-glucoside had the weakest association of −3.26 kcal/mol with MOE. Silvestrol, however, has shown an interaction that binds strongly with MOE and AutoDock Vina, with binding affinities of −7.79 kcal/mol and −7.5 kcal/mol, respectively, hence appearing to be a promising lead compound to study further.

**TABLE 4:** Docking results of the 10 antiviral phytochemicals against the SARS-CoV-2 spike protein receptor binding domain (PDB ID: 7DQA).

Sr	Compound	Vina		MOE	
		RSMD	Binding affinity (Kcal/mol)	RSMD	Binding affinity (Kcal/mol)
1	Castanospermine	0.00	−5.4	0.6138	−3.95
2	Curcumin	0.00	−6.4	1.3344	−6.48
3	Emetine	0.00	−7.7	1.4078	−6.95
4	Emodin-8-glucoside	0.00	−8.0	0.8739	−3.26
5	Ginkgolic acid	0.00	−4.9	2.1399	−6.27
6	Lycorine	0.00	−6.9	1.0826	−5.00
7	Moronic acid	0.00	−8.1	1.4956	−5.46
8	Patentiflorin A	0.00	−8.3	1.2792	−8.85
9	Quercetin	0.00	−7.4	1.0132	−6.26
10	Silvestrol	0.00	−7.5	1.8454	−7.79

Results on molecular interaction between Silvestrol and the SARS-CoV-2 spike protein were investigated in more detail and shown in Table 5. The survey also showed that Silvestrol formed numerous hydrogen bonds with essential residues, such as ASP350, HIS378, HIS401, HIS505, and ARG514, in the system, with bond lengths ranging from 2.18 Å to 2.96 Å, indicating stable interactions. Furthermore, Silvestrol exhibited a hydrophobic relationship with TYR510 and PHE504, which also supports the longevity of the receptor-ligand complex. The interactions play a crucial role in the compound’s ability to bind to the receptor and prevent viral entry.

**TABLE 5:** Molecular interactions of Silvestrol with the SARS-CoV-2 spike protein receptor.

Residue	Bond category	Bond types	From chemistry	To chemistry	Bond length (Å)
ASP350	Hydrogen Bond	Conventional Hydrogen Bond	H-Donor	H-Acceptor	2.41868
ASP350	Hydrogen Bond	Conventional Hydrogen Bond	H-Donor	H-Acceptor	2.17778
HIS378	Hydrogen Bond	Conventional Hydrogen Bond	H-Donor	H-Acceptor	2.95657
HIS401	Hydrogen Bond	Conventional Hydrogen Bond	H-Donor	H-Acceptor	2.87885
HIS505	Hydrogen Bond	Conventional Hydrogen Bond	H-Donor	H-Acceptor	2.86577
ARG514	Hydrogen Bond	Conventional Hydrogen Bond	H-Donor	H-Acceptor	2.2333
ALA348	Hydrogen Bond	Conventional Hydrogen Bond	H-Donor	H-Acceptor	2.88639
ALA348	Hydrogen Bond	Conventional Hydrogen Bond	H-Donor	H-Acceptor	2.69775
ALA348	Hydrogen Bond	Conventional Hydrogen Bond	H-Donor	H-Acceptor	2.23088
TRP349	Hydrogen Bond	Conventional Hydrogen Bond	H-Donor	H-Acceptor	2.31814
TRP349	Hydrogen Bond	Carbon Hydrogen Bond	H-Donor	H-Acceptor	3.54393
GLU406	Hydrogen Bond	Carbon Hydrogen Bond	H-Donor	H-Acceptor	3.58664
ASP382	Hydrogen Bond	Carbon Hydrogen Bond	H-Donor	H-Acceptor	2.8769
GLU398	Hydrogen Bond	Carbon Hydrogen Bond	H-Donor	H-Acceptor	3.32871
GLU402	Electrostatic	Pi-Anion	Negative	Pi-Orbitals	4.98428
GLU402	Electrostatic	Pi-Anion	Negative	Pi-Orbitals	3.8815
TYR510	Hydrophobic	Pi-Sigma	C-H	Pi-Orbitals	3.60367
PHE504	Hydrophobic	Pi-Alkyl	Pi-Orbitals	Alkyl	4.80015

Based on its high binding affinity and favorable molecular interactions, the Silvestrol compound was selected as the lead compound for further studies. This decision was made not only because of the strong binding properties but also due to its safety, which is further explored in the ADMET analysis section.

**FIGURE 3:**
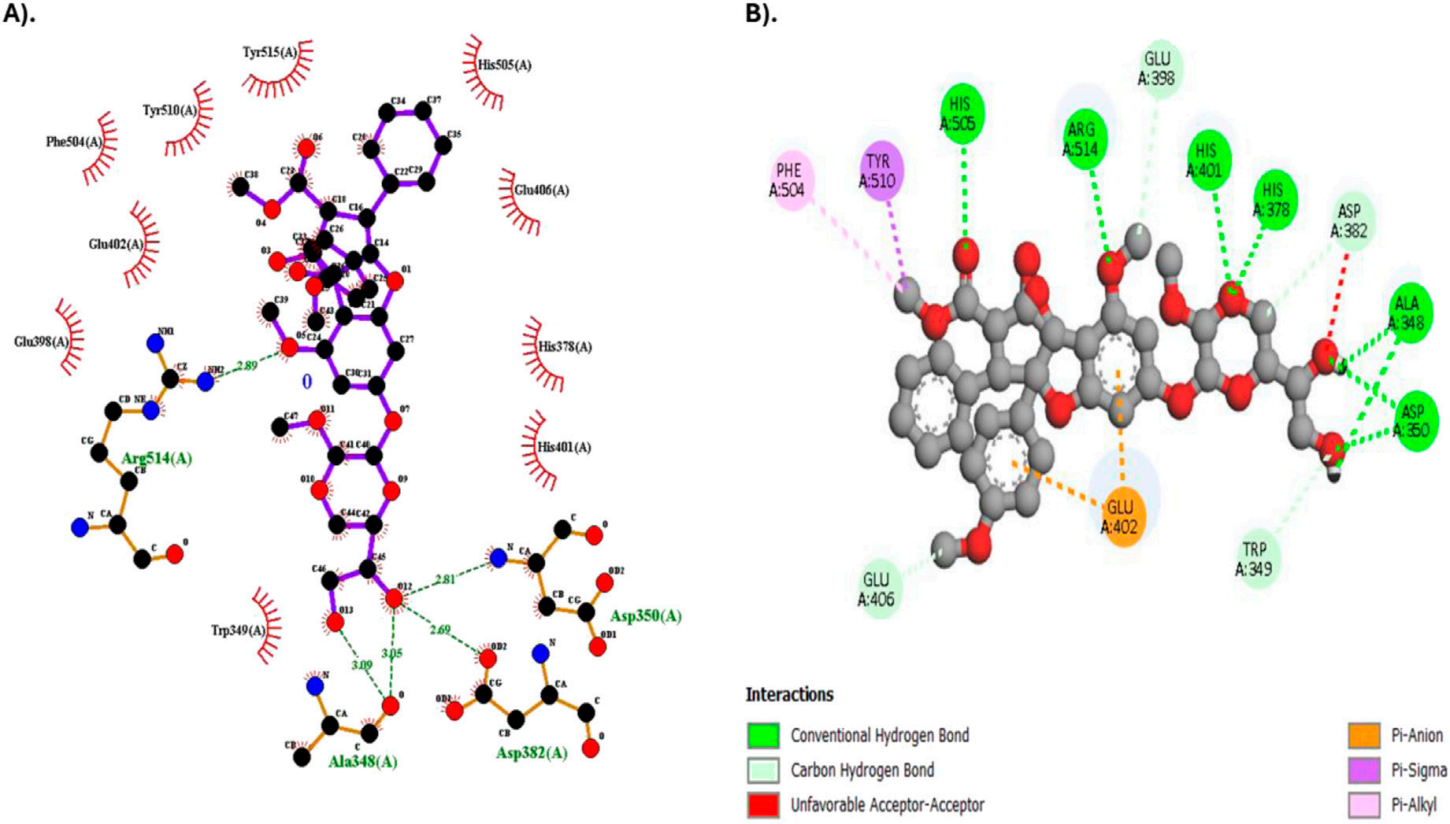
2D Molecular interaction diagram of Silvestrol with the SARS-CoV-2 spike protein receptor. **(A)** Diagram generated using LigPlot+. **(B)** Diagram generated using Discovery Studio.

**FIGURE 4:**
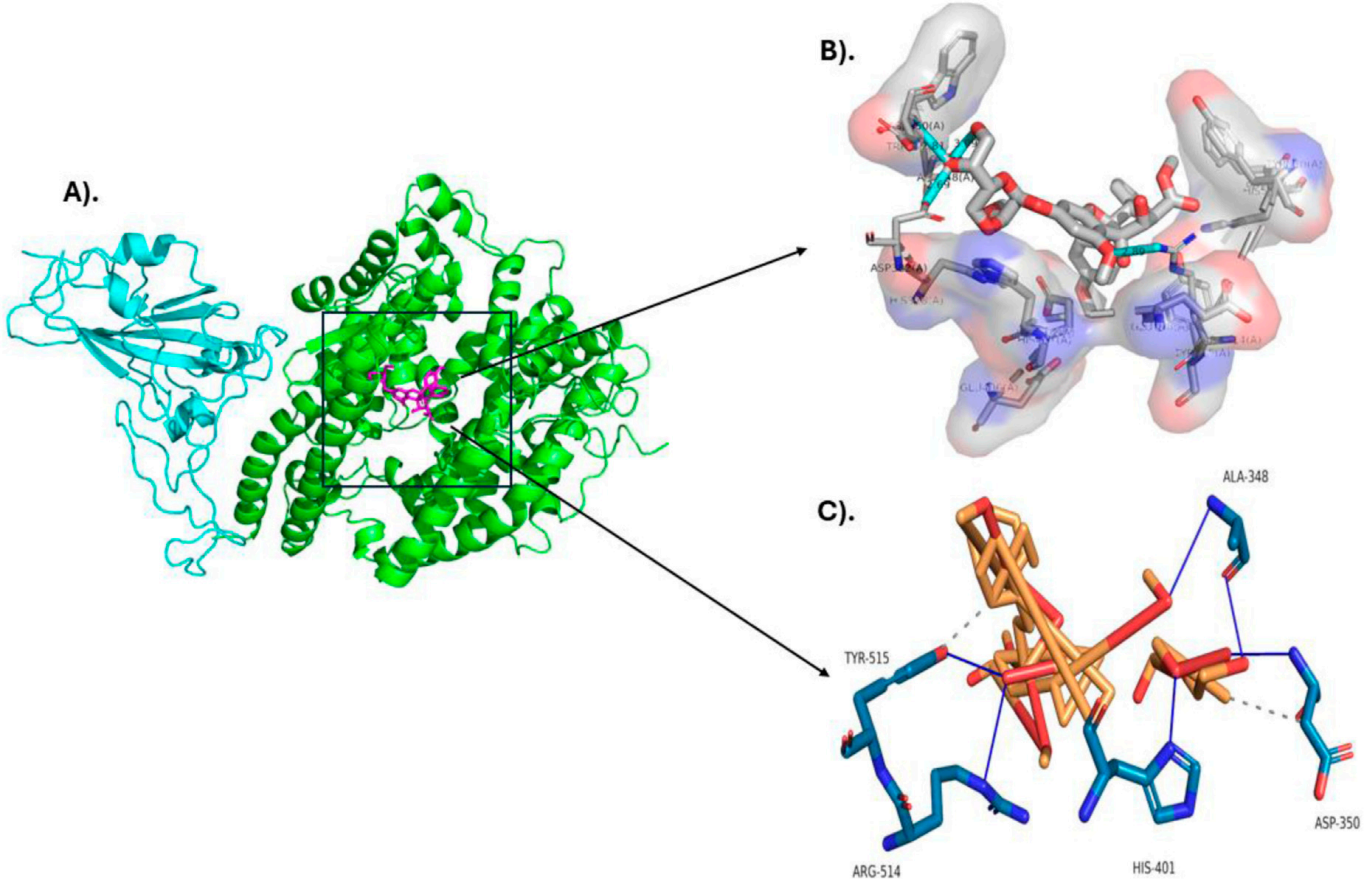
3D Molecular interaction diagram of Silvestrol with the SARS-CoV-2 spike protein receptor. **(A)** 3D dock pose of Silvestrol, **(B)** PyMOL illustration of hydrophobic and hydrogen bonding sites. **(C)** 3D molecular interactions of Silvestrol with the receptor, analyzed using PLIP.

Figure 3 presents a two-dimensional (2D) molecular interactivity plot. Figure 3A is a representation prepared by LigPlot+, whereas Figure 3B is in Discovery Studio. 3D molar interaction is presented in Figure 4, where the 3D dock pose is explained in Figure 4A, PyMOL interpretation of hydrophobic and Hydrogen bonding sites is explained in Figure 4B, and interpretation of 3D moles in terms of molar interactions using PLIP (Protein-Ligand Interaction Profiler) is explained in Figure 4C.

### ADMET screening of phytochemicals

The performance of Silvestrol in the ADMET profile was analyzed using SwissADME and ProTox 3.0, providing a glimpse of its physicochemical properties, pharmacokinetics, druglikeness, and toxicity profile. The physicochemical characteristics of Silvestrol reveal that it is categorized with a molecular weight of 654.66 g/mol and 47 heavy atoms, wherein a reasonable portion of sp3 hybridized carbons has a value of 0.44. This compound has 11 rotatable bonds, 13 hydrogen-bond acceptors, and four hydrogen-bond donors. It also has a molar refractivity of 161.84 and a topological polar surface area (TPSA) of 171.83 Å^2^, which indicates a balanced structure suitable for drug development. Silvestrol has a log P of 1.91, indicating moderate lipophilicity, and its water solubility is classified as moderately soluble, with a log S of −4.49.

**FIGURE 5:**
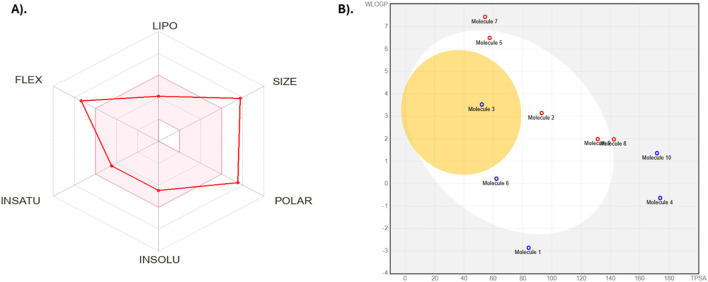
ADMET analysis visualizations of Silvestrol. **(A)** Radar diagram representing the overall ADMET profile of Silvestrol, highlighting its druglikeness and pharmacokinetics. **(B)** Boiled egg diagram illustrating the compound’s solubility and permeability characteristics.

The pharmacokinetics of Silvestrol are predicted to be low in terms of gastrointestinal absorption and unable to be perfused across the blood-brain barrier (BBB). It is a substrate of P-glycoprotein (P-gp), which can pump it out of the cells. It is also an inhibitor of CYP3A4, which can interact with other drugs that are metabolized by this enzyme. Silvestrol does not appear to inhibit the CYP1A2, CYP2C19, CYP2C9, or CYP2D6 enzymes, meaning that it is a potential drug that does not interact with drugs metabolized by those enzymes. Although these properties indicate that Silvestrol does not violate the five rules of Lipinski, it fails to meet Lipinski requirements, as it exceeds the weight limit of 500 g/mol and has more than 10 hydrogen bond donors. Nevertheless, these violations do not imply that Silvestrol cannot become a possible drug, because, in this case, the compound still has good ADMET properties. ADME properties are presented in Table 6; Figure 5.

**TABLE 6:** ADME parameters of Silvestrol retrieved from SwissADME.

ADME parameters	
Physiochemical properties	
Formula	C34H38O13
Molecular weight	654.66 g/mol
Num. heavy atoms	47
Num. from. Heavy atoms	18
Fraction Csp3	0.44
Num. rotatable bonds	11
Num. H-bond acceptors	13
Num. H-bond donors	4
Molar Refractivity	161.84
TPSA	171.83 Å (Molloy et al., 2023)
Lipophilicity	
Log Po/w	1.91
Water Solubility	
Log *S* (ESOL)	−4.49
Class	Moderately soluble
Pharmacokinetics	
GI absorption	Low
BBB permeant	No
P-gp substrate	Yes
CYP1A2 inhibitor	No
CYP2C19 inhibitor	No
CYP2C9 inhibitor	No
CYP2D6 inhibitor	No
CYP3A4 inhibitor	Yes
Drug likeness	
Lipinski	No; 2 violations: MW > 500, NorO>10

The toxicity levels of ProTox 3.0 indicate that Silvestrol has a toxicity classification of 5, which is moderate (Table 7). Comparatively, Silvestrol exhibits a much safer toxicity profile than the other eight compounds, several of which have a lower LD50, including Emetine (LD50 = 1 mg/kg) and Patentiflorin A (LD50 = 13 mg/kg). In the toxicity parameters of Silvestrol, the probability values of its active toxicity parameters indicate that Silvestrol has low toxicity in the majority of categories (Table 8). Particularly, Silvestrol exhibits inactive predictions in respect (hepatotoxicity: 0.87; neurotoxicity: 0.84; carcinogenicity: 0.78; mutagenicity: 0.67; cytotoxicity: 0.66; ecotoxicity: 0.58; clinical toxicity: 0.52). Remarkably, immunotoxicity is active with a high probability of 0.99, and toxicity regarding nutrition is active with a probability of 0.63.

**TABLE 7:** Toxicity comparison of Silvestrol with nine other antiviral phytochemicals.

Sr	Compound	LD50 (mg/kg)	Class	Avg. Similarity %	Prediction accuracy %
1	Castanospermine	1,370	4	89.46	70.97
2	Curcumin	2000	4	100	100
3	Emetine	1	1	100	100
4	Emodin-8-glucoside	3,000	5	82.05	70.97
5	Ginkgolic acid	230	3	100	100
6	Lycorine	750	4	100	100
7	Moronic acid	750	4	100	100
8	Patentiflorin A	13	2	95.54	72.0
9	Quercetin	159	3	100	100
10	Silvestrol	2,300	5	64.71	68.07

**TABLE 8:** Toxicity profile of Silvestrol from ProTox 3.0.

Classification	Target	Prediction	Probability
Organ toxicity	Hepatotoxicity	Inactive	0.87
	Neurotoxicity	Inactive	0.84
	Nephrotoxicity	Active	0.61
	Respiratory toxicity	Active	0.68
	Cardiotoxicity	Active	0.65
Toxicity end points	Carcinogenicity	Inactive	0.78
	Immunotoxicity	Active	0.99
	Mutagenicity	Inactive	0.67
	Cytotoxicity	Inactive	0.66
	BBB-barrier	Inactive	0.7
	Ecotoxicity	Inactive	0.58
	Clinical toxicity	Inactive	0.52
	Nutritional toxicity	Active	0.63
Tox21-Nuclear receptor signaling pathways	Aryl hydrocarbon Receptor (AhR)	Inactive	0.82
	Androgen Receptor (AR)	Inactive	0.98
	Androgen Receptor Ligand Binding Domain (AR-LBD)	Inactive	0.89
	Aromatase	Inactive	0.75
	Estrogen Receptor Alpha (ER)	Inactive	0.85
	Estrogen Receptor Ligand Binding Domain (ER-LBD)	Inactive	0.93
	Peroxisome Proliferator Activated Receptor Gamma (PPAR-Gamma)	Inactive	0.78
Tox21-Stress response pathways	Nuclear factor (erythroid-derived 2)-like 2/antioxidant responsive element (nrf2/ARE)	Inactive	0.86
	Heat shock factor response element (HSE)	Inactive	0.86
	Mitochondrial Membrane Potential (MMP)	Inactive	0.67
	Phosphoprotein (Tumor Supressor) p53	Active	0.61
	ATPase family AAA domain-containing protein 5 (ATAD5)	Inactive	0.86
Molecular Initiating Events	Thyroid hormone receptor alpha (THRα)	Inactive	0.68
	Thyroid hormone receptor beta (THRβ)	Active	0.77
	Transthyretin (TTR)	Inactive	0.59
	Ryanodine receptor (RYR)	Inactive	0.82
	GABA receptor (GABAR)	Inactive	0.67
	Glutamate N-methyl-D-aspartate receptor (NMDAR)	Inactive	0.98
	Alpha-amino-3-hydroxy-5-methyl-4-isoxazolepropionate receptor (AMPAR)	Inactive	0.99
	Kainate receptor (KAR)	Inactive	0.99
	Acetylcholinesterase (AChE)	Inactive	0.71
	Constitutive androstane receptor (CAR)	Inactive	0.99
	Pregnane X receptor (PXR)	Inactive	0.54
	NADH-quinone oxidoreductase (NADHOX)	Inactive	0.62
	Voltage gated sodium channel (VGSC)	Active	0.91
	Na+/I- symporter (NIS)	Inactive	0.78
Metabolism	Cytochrome CYP1A2	Inactive	0.97
	Cytochrome CYP2C19	Inactive	0.76
	Cytochrome CYP2C9	Inactive	0.56
	Cytochrome CYP2D6	Inactive	0.72
	Cytochrome CYP3A4	Inactive	0.73
	Cytochrome CYP2E1	Inactive	0.97

Additionally, the pathway phosphoprotein (tumor suppressor) p53, which belongs to the class of stress response proteins, is also predicted to be active with a 0.61 probability. These findings demonstrate that Silvestrol is relatively safe, but specific concerns remain regarding immune and nutritional toxicity, and thus, it should be further studied. According to toxicity and ADMET screening, Silvestrol is a compound that exhibits more desirable drug-like properties compared to other antiviral phytochemicals in the present research. It has a good physicochemical profile, moderate lipophilicity, and a desirable toxicity level (class 5, LD50 = 2,300 mg/kg), making it a potential choice for further investigation. However, as promising as these outcomes are, they must be further tested in an *in vivo* trial to ensure their safety and efficacy prior to use in any clinical scenario.

### Molecular dynamics (MD) simulation and MMGBSA analysis of receptor protein and lead antiviral compound

**FIGURE 6:**
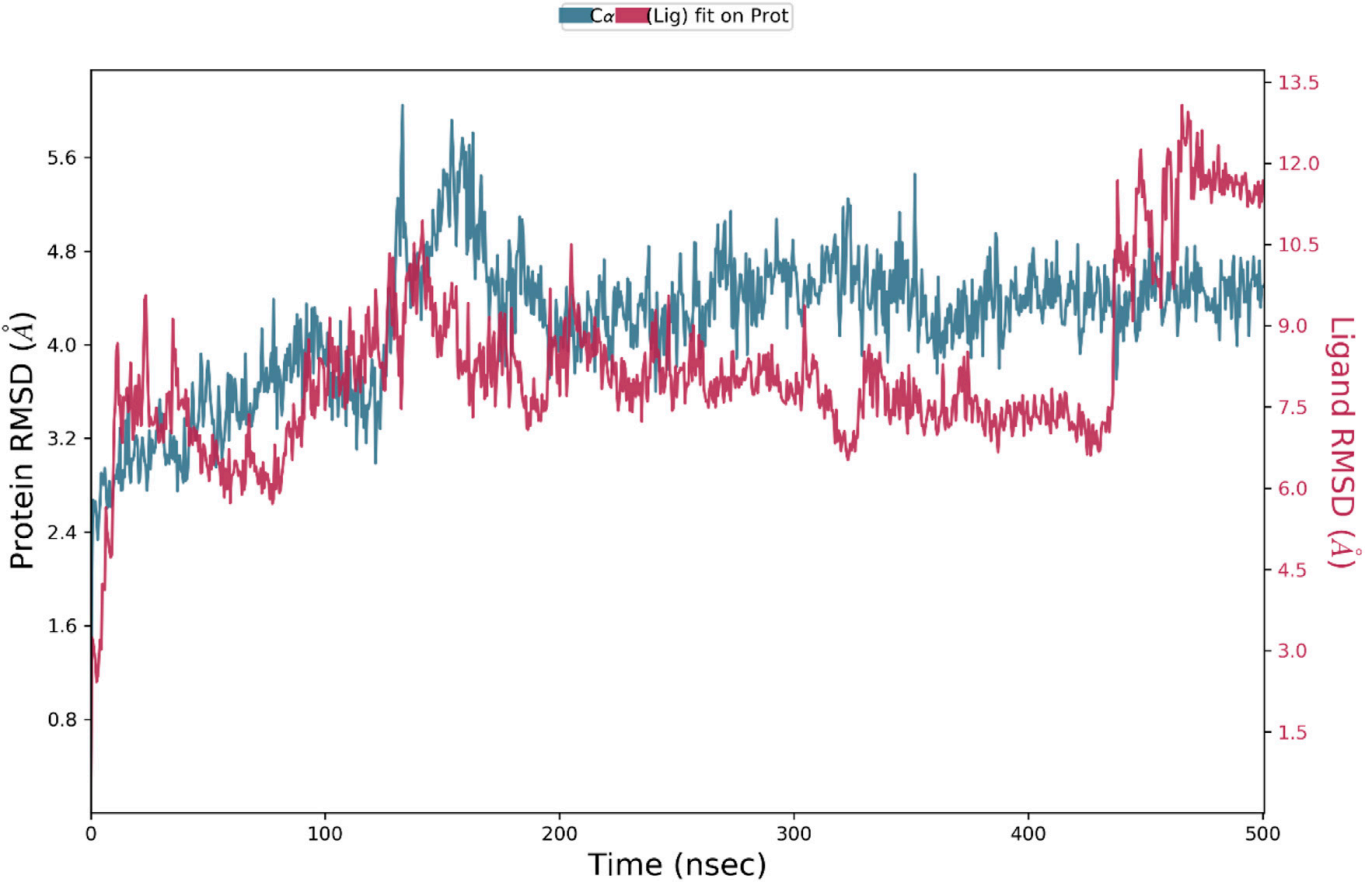
Protein-Ligand RMSD shows deviations of protein and ligand RMSD over 500 ns, stabilizing after 150 ns.

The protein RMSD displays variations during the course of the simulation of 500 ns (Figure 6). The RMSD stabilizes around 150 ns, when deviations in the structure of the protein and stay between 2 and 3 Å. These fluctuations are indicative of slight conformational dynamical movements in the protein structure at the initial steps of the simulation, then a stabilization as the system balanced-out. As far as Ligand RMSD is concerned there is a lot of variation at the start of the simulation and the ligand has more variance particularly in the first 150 ns Once this duration has passed the RMSD on the ligand settles at approximately 56 Å. The oscillations imply that the ligand adapts to the binding pocket in the first place and then stabilized in the pocket, which implies formation of a more permanent interaction with the protein. This indicates that; as the ligand is subjected to some conformational changes, it will finally fit into a stable orientation in the binding pocket.

**FIGURE 7:**
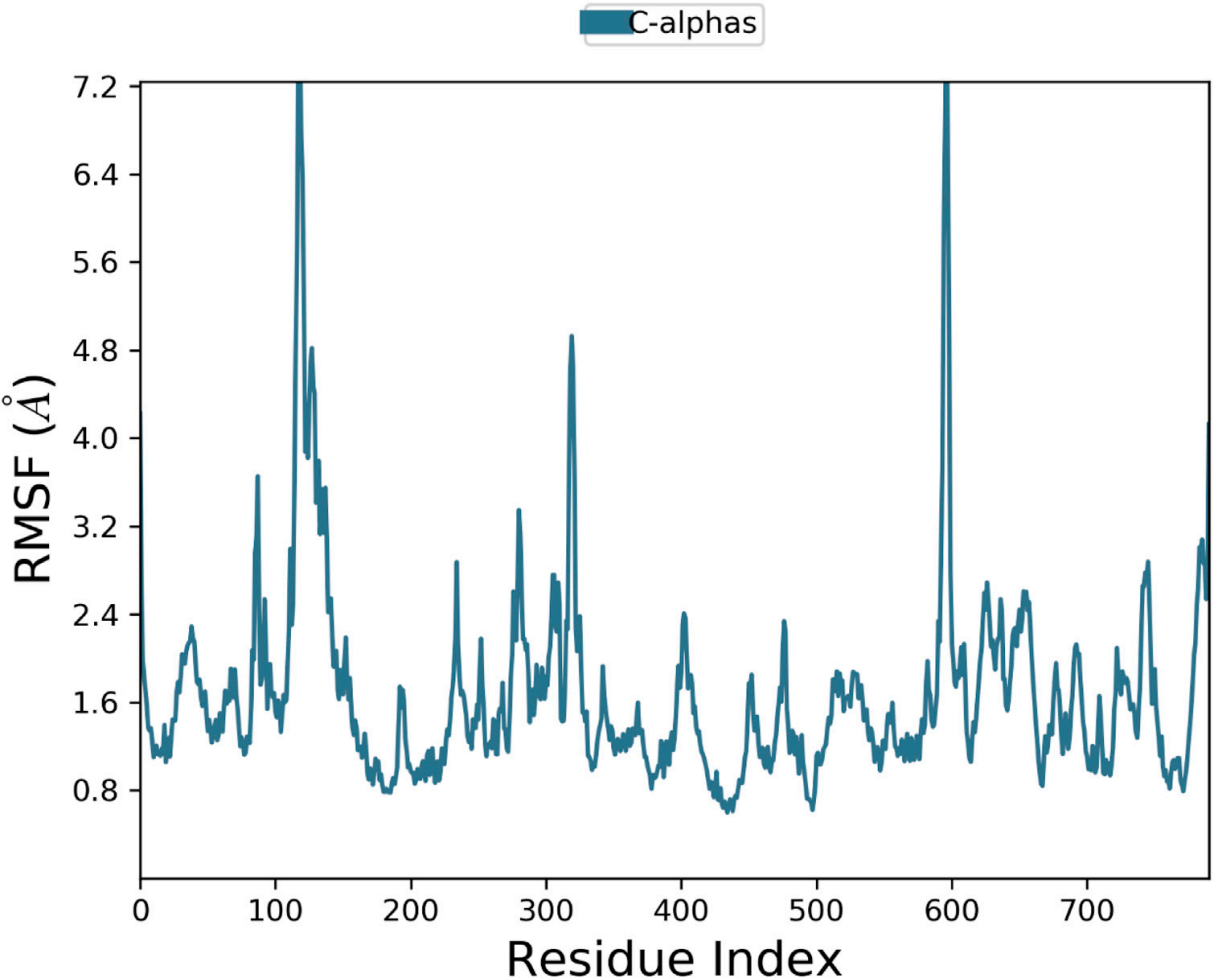
Protein RMSF flexibility throughout the simulation, with higher fluctuations in specific regions.

In the RMSF plot (Figure 7), shows flexibility the protein was throughout the simulation. This is because there are high values of RMSF in certain parts of the protein especially at 100–150, 300–350 and 600–650 suggesting that the protein is flexible in these areas. These mobile sections could be important during the protein-ligand interaction. The comparably large flexibility of some of the regions implies that such regions of the protein may be the dynamic interfaces, which may add to the binding of the ligand or stability of the ligand in the pocket.

**FIGURE 8:**
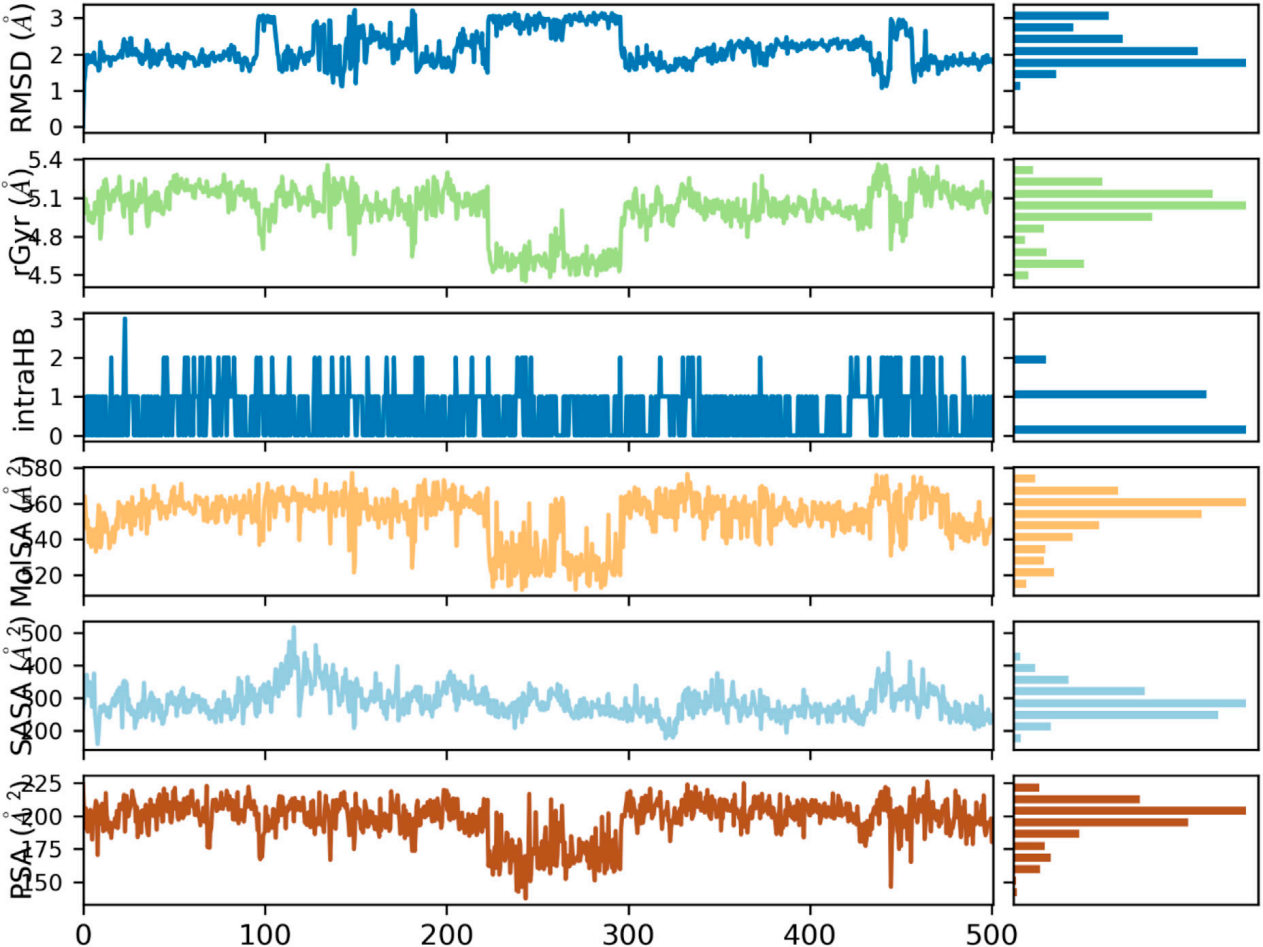
RSMD, rGyr, IntraHB, MolSA, SASA and PSA of ligand throughout 500ns simulation.

RMSD (Root Mean Square Deviation) of both the protein and the ligand depicts their structural stability with regard to the simulation. This RMSD of the protein becomes stable within 150 ns and varies around 2 A, which portrays that once the protein undergoes the initial conformational adaptations, it acquires the stable configuration. By comparison, ligand RMSD is more sensitive to fluctuations initially, reaching equilibrium state at 5–6 Å after 150 ns. This implies that the ligand undergoes largely conformational fitting in the binding pocket and subsequently establishing in a certain orientation. The ligand-protein complex in itself is represented by the rGyr (Radius of Gyration) which indicates its compactness. This value of rGyr fluctuates within the range between 4.5 and 5.0 Å and has a slight change during the simulation. This shows that there is no marked expansion or contraction in the overall outline of the complex; hence suggestion lies in the fact that the complex is quite compact in nature and has received its build structure over the years. The IntraHB (Intramolecular Hydrogen Bonds) graph depicts variability in the quantity of hydrogen bonds created as intramolecularly within the ligand. Such oscillations indicate that the ligand retains certain level of internal stability, as during the simulation it averaged 2-3 hydrogen bonds. These bonds are important to stabilize the structure of ligand so that it could maintain its conformation at binding pocket and thereby add to overall stability of ligand-protein interaction. The MolSA (Molecular Solvent Accessible Surface Area) and SASA (Solvent Accessible Surface Area) indicates to what extent the ligand and protein surface are solvent accessible. The two properties start with a lot of fluctuation at the beginning of the simulation but later achieves stability after 150 ns. MolSA becomes stable approximately between 20–25 and 20–25 Å^2^ and the SASA becomes stable at about 50–60 Å^2^. This shows that as simulation unfolds, the ligand is buried more in the binding pocket and less exposed to the solvent to develop a stronger binding interaction with the protein. These values indicate that the ligand is properly inserted into a binding site of the protein and a new complex is stable and has a favorable energy. The hydrophilic characteristic of ligand can be reflected through PSA (Polar Surface Area) which indicates the exposure of polar surface area of ligand. The PSA values go up and down between the simulation but converge towards 175–200 Å^2^ meaning that the ligand has a constant state of polarity and it might bind to the active site of the protein through hydrogen bonding and electrostatic interactions. All ligand properties graphs can be seen in Figure 8.

**FIGURE 9:**
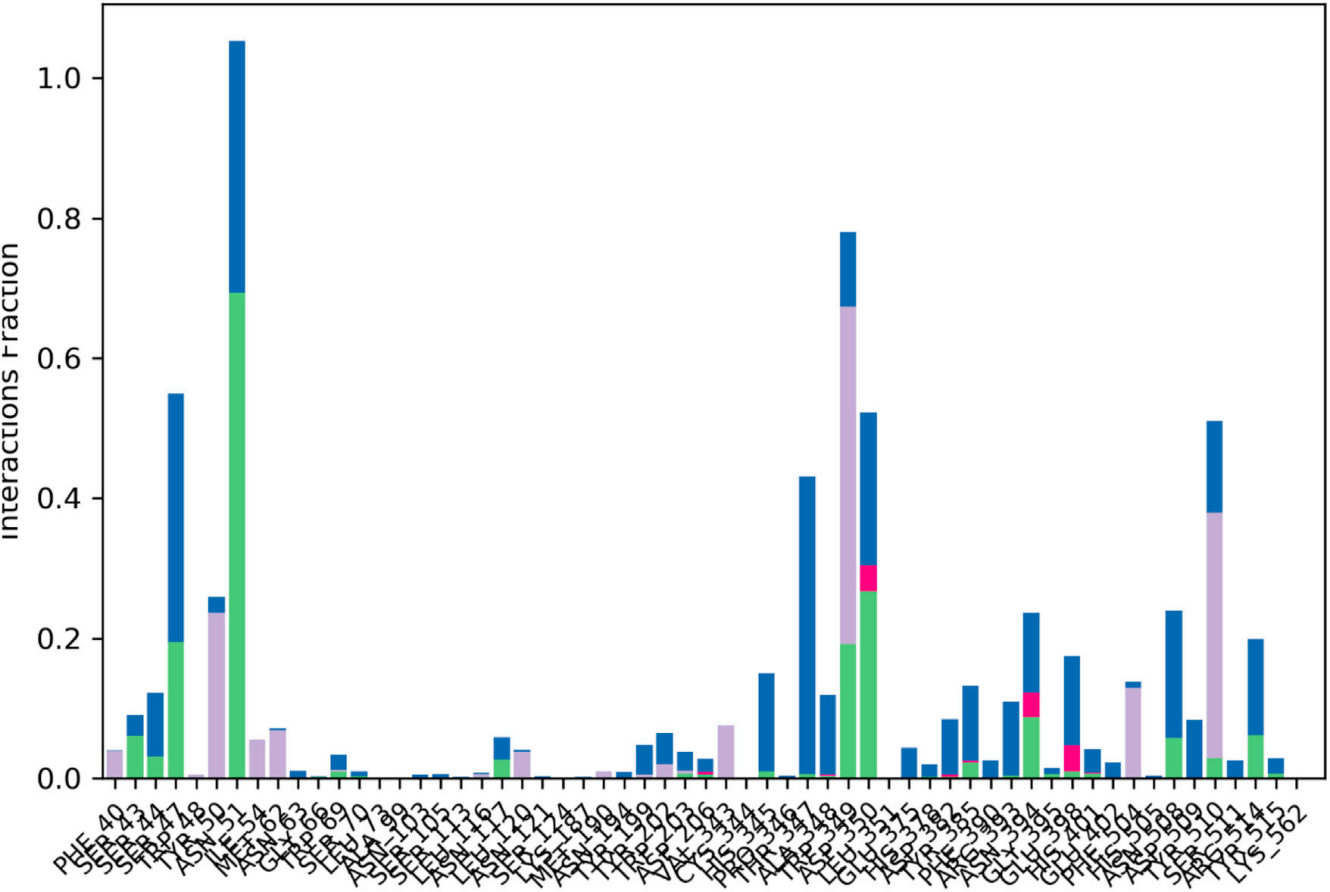
Protein-Ligand Contact Histogram frequency of interactions between the protein and ligand, highlighting hydrophobic, hydrogen bonding and salt bridges interactions.

Protein-Ligand Contact Histogram which reflects the interactions frequency between the protein and the ligand (Figure 9). Remarkably, a number of peaks depicting that the ligand and certain residues of the protein interact have been observed. As indicated by the blue and green bars, the main category of interactions is the hydrophobic and hydrogen bonding contact, and the pink and purple bars indicate electrostatic interactions. Such fractions of interaction indicate a highly interacted ligand in the pocket with strong binding network with the protein.

**FIGURE 10:**
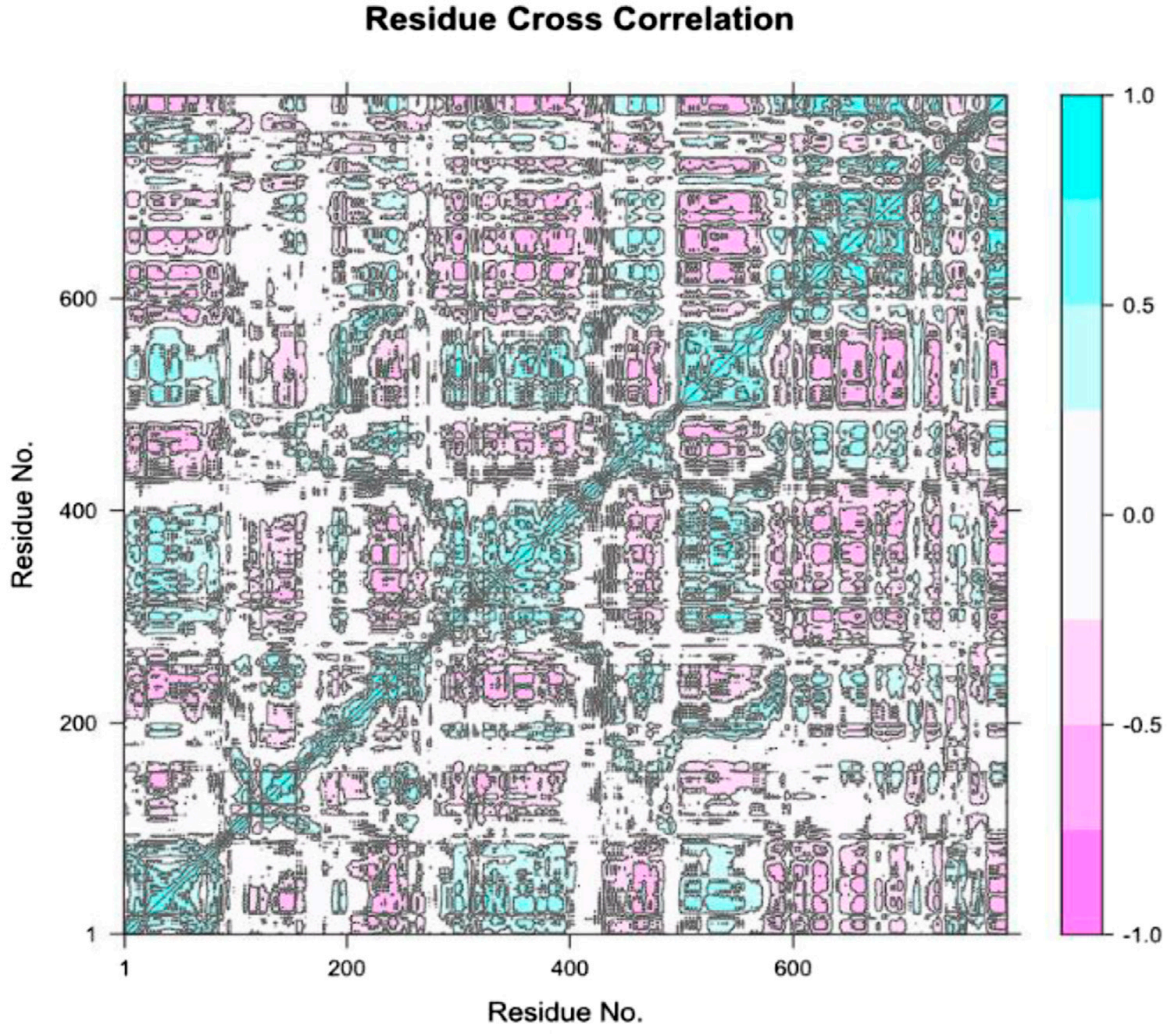
Residue Cross-Correlation Map (DCCM) showing the correlated motions between different residues of the protein. The plot highlights areas of strong positive and negative correlations, suggesting key regions involved in protein function and potential ligand binding sites.

The Residue Cross-Correlation Map (DCCM), the diagram describes the motions that occur in different parts of the protein that are correlated to each other (Figure 10). The high diagonal line shows that there are a lot of residues marching to the same beat a fact that is typical of the movement of the proteins in the simulation of dynamics of molecules. The seen color changes of plot, blue and pink regions indicate a strong positive and negative nearest neighbor correlations. This data is crucial when confirming important residues that are possibly part of protein functioning, ligand bindings or conformational alteration. The correlations that appeared to be high in certain areas may indicate functional sites or significant interactions on the protein structure.

**FIGURE 11:**
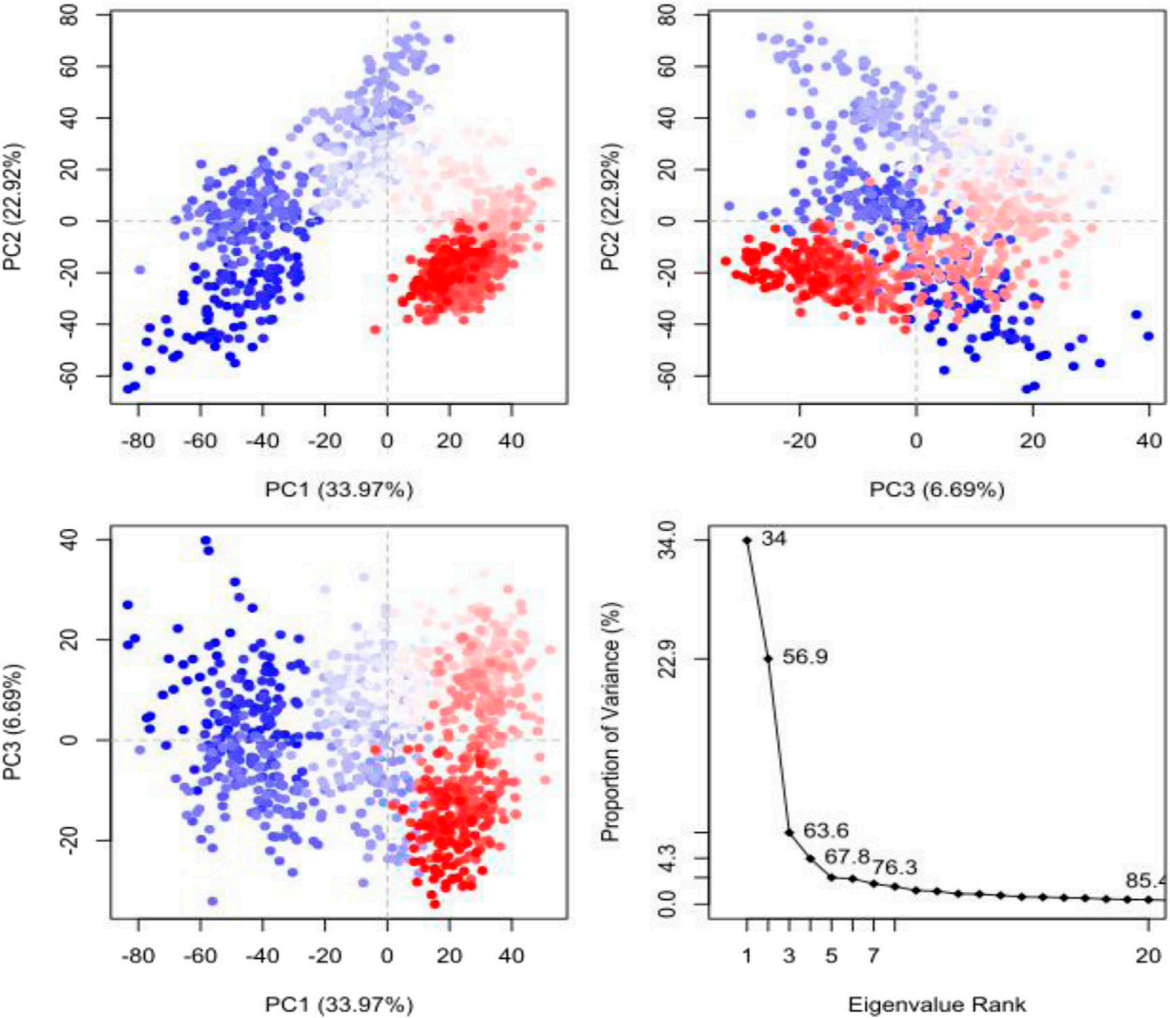
Principal Component Analysis (PCA) of the protein-ligand complex. The scatter plots show the distribution of the first three principal components (PC1, PC2, and PC3), revealing two distinct conformational clusters and the proportion of variance explained by each principal component.

An improvement on our understanding of the motion of the protein ligand complex was given by the Principal Component Analysis (PCA) (Figure 11). There is an indication in the analysis that PC1, PC2, and PC3 are the first three variables that occupy a considerable share of the variance in the system and that PC1 had a variance explained of 33.97 and PC2 22.92. The existence of two clusters of conformations in the scatter plots reveals that the protein experiences significant changes in the conformations during the simulation. The binding of Silvestrol and the following shifts in conformational states are probably related and may indicate that the protein is switching between the stable and unstable states. The proportion of variance plot points out that the initial components are dominant in the system motion and this aspect is critical in description of the protein ligand interaction dynamics.

**FIGURE 12:**
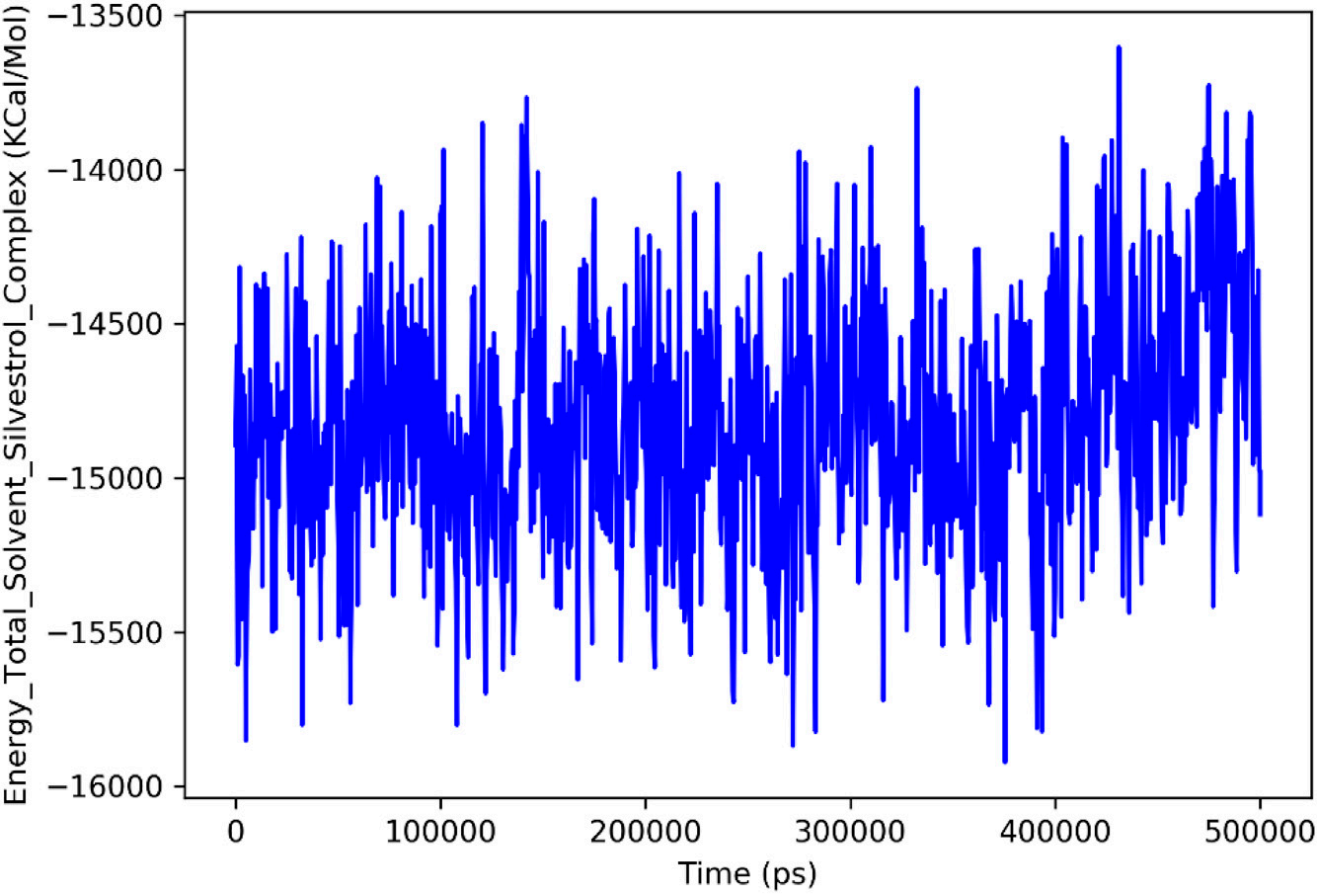
Total Energy Plot of the Silvestrol-Solvent Complex during the MD simulation. The plot shows fluctuations in the total energy of the system over time, with the energy stabilizing after the initial equilibration phase.

The analysis of Silvestrol simulation and SARS-CoV-2 spike protein receptor presented in the post-MD can provide a number of valuable findings regarding the behavior and stability of the system (Figure 12). Going to the Total Energy graph of Silvestrol-Solvent Complex, the variates around −16,000 kcal/mol to −14,500 kcal/mol show that the system was in the stable state after initial equilibration period. These fluctuations indicate that the system has undergone to the steady state and the energy fluctuations are quite normal and typical to a stable molecular dynamics simulation. The significance here is that this stabilization validates the fact that equilibrium state of the simulation is attained that is worthy of subsequent analysis.

Lastly, the MMGBSA analysis which provides the binding free energy of the protein-ligand complex at both of the critical timepoints; contributes to our insights on the stability of the complex. First At a binding energy of 0ns the value is, the interaction of Silvestrol with the SARS-CoV-2 spike protein receptor, is much stronger (−68.56 kcal/mol). At 500 ns however, the binding energy has deteriorated to −25.55 kcal/mol, thus indicating a weakening of the bond with time. The rearrangement of binding energy suggests that the interaction between the protein and the ligand undergoes some sort of disturbance or conformation alteration and hence a reduction in the overall binding strength. This diminishing effect on the interaction is essential to the explanation of stability and possible efficacy of Silvestrol as therapeutic agent.

According to the simulations with MD, Silvestrol binding pocket fits into the SARS-CoV-2 spike protein receptor pocket. It is stable at 150 ns with the protein, whereas the ligand has some conformational changes but after that, it reaches a stable orientation. The initial binding energy the MMGBSA calculation provides is very strong and decays a little bit with time but still provides information of a stable interaction. The Protein-Ligand Contact Histogram, Residue Cross-Correlation Map and PCA also contribute to saying that the ligand creates strong links in the pocket. All in all, the binding pattern affinity of Silvestrol indicates its suitability in the place of a therapeutic agent.

### Pharmacophore characterization and DFT analysis of lead antiviral phytochemical

**FIGURE 13:**
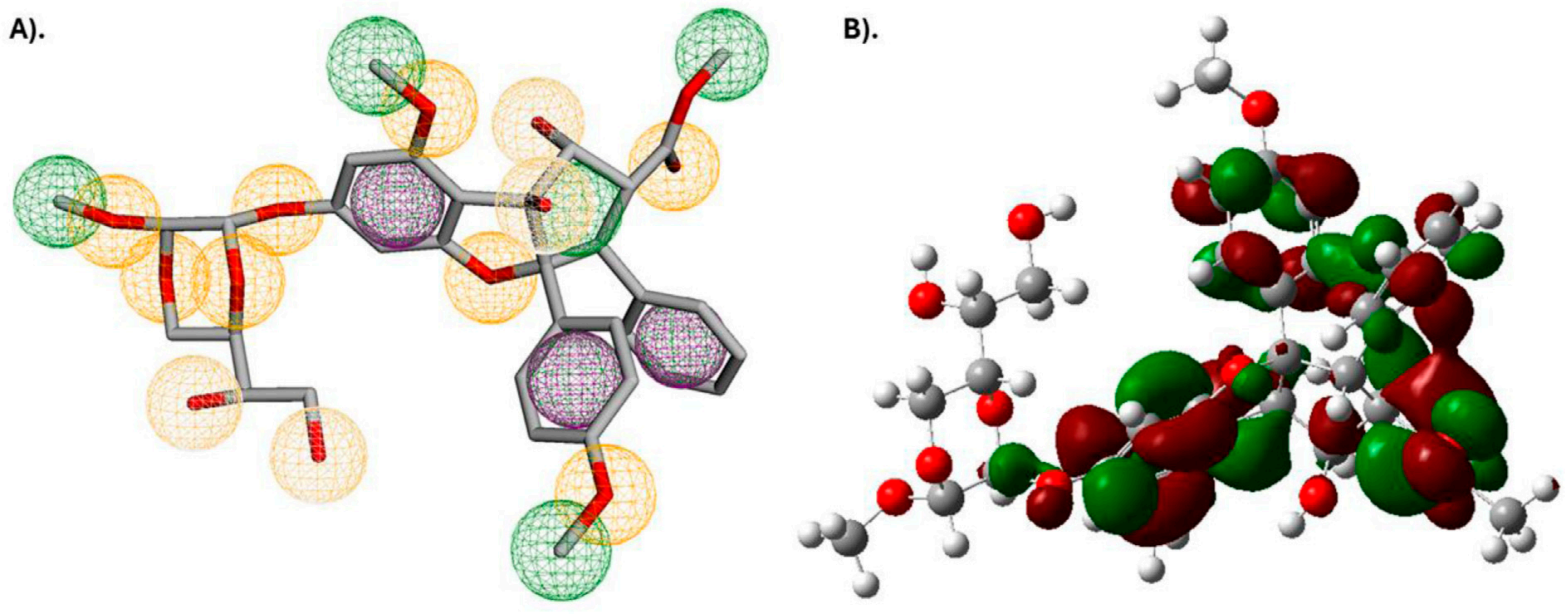
**(A)** Pharmacophore characterization illustration of Silvestrol and **(B)** Provides an illustration of the HOMO and LUMO orbital distributions of Silvestrol, obtained from the DFT calculations. The green and red lobes represent the positive and negative phases of the molecular orbitals, respectively, which are crucial for understanding the electron distribution and reactivity of the compound.

Silvestrol, the lead phytochemical antiviral agent, was characterized with a pharmacophore, which identified the important characteristics that it has in interacting with the SARS-CoV-2 spike protein receptor binding domain. Silvestrol was found to have three aromatic sites, four donor hydrogen atoms, and 12 hydrogen acceptors. It further revealed eight hydrophobic sites, indicating that it will produce strong binding interactions. Figure 13A illustrates the distribution of such pharmacophore sites, and it is apparent that Silvestrol has numerous possible interaction sites, thereby improving its chances of binding to this receptor.

The HOMO-LUMO of Silvestrol was determined through calculations performed in Density Functional Theory (DFT) analysis of the battery, to elucidate the electronic properties of the subject. LUMO was determined to be −0.01031, whereas the HOMO was −0.21637. The computed HOMO LUMO energy gap of Silvestrol is 0.20606 eV based upon the difference between the Highest Occupied Molecular Orbital (HOMO) = −0.21637 eV and the Lowest Unoccupied Molecular Orbital (LUMO) = −0.01031 eV. This small energy gap indicates that Silvestrol is quite reactive, as a smaller gap between HOMO and LUMO typically correlates with higher chemical reactivity. These compounds are more easily involved in electronic transitions, making them good targets for biological ones. Within drug design, a smaller energy gap is associated with improved binding affinity and potency.

In addition, HOMO should be considered a representation of the molecule’s ability to donate electrons. In contrast, LUMO should be considered to be the ability of the molecule to accept electrons. The close spacing of these energy states in Silvestrol suggests an effective electron transfer potential, which is a favorable property in terms of reactivity with biomolecular targets (Figure 13B). The HOMO-LUMO energy gap of Silvestrol accentuates its capability as a reactive and suitable lead compound for use in antiviral drug development.

## Discussion

Currently, the COVID-19 disease, which is caused by SARS-CoV-2, has primarily affected the health of all people worldwide, as children are more prone to this disease due to a shortage of therapeutic interventions. Existing remedies for pediatric COVID are scarce, and current antiviral treatments can pose safety concerns. The goal of the study was to investigate the potential use of Silvestrol, a natural compound produced by the species Aglaia, as a therapeutic agent for pediatric COVID-19 ^37^.

Based on the currently reported molecular docking research, Silvestrol displays a powerful binding affinity to the receptor-binding domain (RBD) of the SARS-CoV-2 spike protein, associated with a binding energy of −7.5 kcal/mol. This compares with studies conducted by Grunweller et al. (2018), who found that Silvestrol decreases viral expression by targeting the eIF4A component of the viral replication system (Müller et al., 2018). Its docking also displayed vital interactions of the Silvestrol with important substitutions in the RBD, confirming its usefulness as a good inhibitor (Pillaiyar et al.). The ADMET modeling and analysis showed that Silvestrol has a good pharmacokinetics profile (moderate PRS and PRC). It has a low toxicity profile with an LD50 of 2,300 mg/kg, belonging to the toxicity class 5, which indicates a significant safety margin. These results are consistent with reports of the compound’s non-toxicity in various *in vitro* models (Thomas et al., 2021).

Molecular dynamics (MD) simulations Silvestrol-receptor-binding domain (RBD) of the SARS-CoV-2 spike protein have offered valuable information in the dynamic motion of the protein-ligand complex. Root Mean Square Deviation (RMSD) of protein exhibited some short-term variations during the first 150 ns, but then it stabilized somewhere around 2–3 Å, which was indicating that the protein has made some slight conformational changes before stabilizing in one particular conformation. On the other hand, ligand RMSD showed great changes in the early stage of the simulation process, especially within the first 150 ns, followed by stabilizing to approximately 5–6 A, which implied that the ligand became adaptable to the binding pocket and achieved the stable spatial position. This tendency is consistent with the prior observations that ligand binding is most times followed by the adjustment of various actions of the same prior to the stable interactions with other proteins.

The molecular dynamics (MD) results collectively indicate that silvestrol maintains a stable and biologically plausible interaction with the SARS-CoV-2 spike protein receptor-binding domain. The stabilization of RMSD values after 150 ns, combined with a consistent radius of gyration and a reduction in solvent-accessible surface area, suggests that silvestrol becomes firmly embedded within the binding pocket over time. This behavior reflects not only structural compatibility but also the likelihood of sustained receptor engagement *in vivo*. The persistence of hydrogen bonding and hydrophobic contacts with key residues (e.g., ASP350, HIS378, TYR510) reinforces the compound’s ability to form durable interactions critical for antiviral activity. Furthermore, principal component analysis confirmed that the protein–ligand complex occupies well-defined conformational states, consistent with stable binding dynamics. Taken together, these findings imply that silvestrol could maintain its inhibitory effect under physiological conditions, making it a promising candidate for pediatric antiviral therapy despite natural fluctuations in binding free energy during the simulation. Although the MM-GBSA binding energy decreased from −68.56 kcal/mol at 0 ns to −25.55 kcal/mol at 500 ns, this change reflects the natural relaxation of the complex rather than a loss of interaction. Molecular dynamics simulations consistently showed stable structural parameters, including RMSD stabilization of both protein and ligand after 150 ns, maintenance of compact rGyr values, and reduced SASA indicating deeper ligand embedding. Furthermore, persistent hydrogen bonds and hydrophobic interactions with key residues were observed throughout the simulation, while PCA revealed stable conformational states. Taken together, these complementary findings indicate that Silvestrol remains stably bound within the SARS-CoV-2 spike protein receptor pocket, supporting its potential efficacy despite the relaxation in MM-GBSA energy values.

The density functional theory (DFT) analysis provides additional insight into silvestrol’s chemical reactivity and binding potential. The small HOMO-LUMO energy gap (0.206 eV) reflects high electronic reactivity, suggesting that silvestrol can effectively participate in charge transfer interactions with amino acid residues in the spike protein binding pocket. This property may facilitate the formation of stable hydrogen bonds and electrostatic interactions, thereby strengthening its inhibitory activity (Musarra-Pizzo et al., 2021). From a translational perspective, the favorable electronic profile of silvestrol enhances its suitability as a lead compound, as such reactivity often correlates with improved biological activity against viral targets. These results support the MD findings by offering a mechanistic explanation for silvestrol’s stable interactions, further validating its potential as a therapeutic agent in pediatric COVID-19. Although Silvestrol demonstrates violations of Lipinski’s rule of five and shows low gastrointestinal absorption, such limitations are not unusual among clinically used natural products and complex antivirals. Paclitaxel, amphotericin B, and remdesivir all share poor oral bioavailability or permeability properties yet remain approved and widely used through intravenous or optimized formulations (Fernández-García and Fraguas-Sánchez, 2024; Chen et al., 2021; Sze et al., 2019). These precedents suggest that similar pharmaceutical strategies could be applied to Silvestrol, particularly for pediatric delivery, where safety and controlled administration via non-oral routes may be more feasible. Therefore, while pharmacokinetic barriers exist, they do not negate Silvestrol’s therapeutic potential but instead highlight the need for formulation innovation and targeted delivery approaches.

Although Patentiflorin A achieved the most favorable docking affinity (−8.85 kcal/mol), its predicted toxicity profile was concerning, with an LD_50_ of 13 mg/kg and classification in toxicity class 2. Such a profile indicates a high likelihood of adverse effects, rendering it unsuitable for pediatric therapeutic exploration. By comparison, Silvestrol demonstrated a slightly lower binding affinity (−7.5 kcal/mol) but was predicted to have a significantly safer toxicity profile, with an LD_50_ of 2,300 mg/kg and classification in toxicity class 5. This safety margin, combined with its broad-spectrum antiviral potential and established pharmacological data, provided a strong rationale for prioritizing Silvestrol in downstream ADMET evaluation, molecular dynamics simulations, and pharmacophore modeling. Given the intended focus on pediatric applications, where tolerability and safety are of paramount importance, the selection of Silvestrol over Patentiflorin A was both scientifically and clinically justified.

While this study is based exclusively on *in silico* methodologies including molecular docking, dynamics simulation, pharmacophore mapping, ADMET profiling, and DFT analysis these approaches serve to generate hypotheses and prioritize candidates in early-stage antiviral screening. Nevertheless, several peer-reviewed studies have provided compelling *in vitro* and *in vivo* evidence that supports the translational relevance of silvestrol. In particular, Müller et al. (2017) demonstrated potent inhibition of MERS-CoV and HCoV-229E replication in human primary cells at low nanomolar concentrations (EC_50_ values of ∼1.3 nM and 3 nM, respectively) without notable cytotoxicity (Müller et al., 2018). Similarly, Obermann et al. (2022) confirmed silvestrol’s efficacy in inhibiting SARS-CoV-2 translation in cell-based assays using dual-luciferase systems (Obermann et al., 2022). Additionally, Schiffmann et al. (2022) characterized silvestrol’s *in vitro* safety and bioavailability, showing minimal off-target effects, no mutagenicity in Ames or micronucleus assays, and good stability in liver microsomes at nanomolar levels (Schiffmann et al., 2022). Furthermore, Todt et al. (2018) demonstrated that silvestrol potently inhibits hepatitis E virus (HEV) replication both *in vitro* and *in vivo* in humanized mouse models, revealing a promising antiviral effect beyond coronaviruses (Todt et al., 2018). Finally, Blum et al. (2020) reported broad-spectrum antiviral activity of silvestrol across a range of RNA viruses such as Ebola, Zika, Chikungunya, and picornaviruses underscoring its general antiviral potential and relevance to our computational observations (Blum et al., 2020). These experimental findings, collectively, lend strong support to our computational results and reinforce the rationale for further experimental validation. In the revised manuscript, we have clearly emphasized this limitation in the Conclusions section and highlighted these literature-backed precedents to contextualize silvestrol’s candidacy as a pediatric antiviral lead.

Pharmacokinetic and safety profiles in children differ substantially from those in adults, and that our current study does not address these pediatric-specific differences. Developmental variations in absorption, distribution, metabolism, and excretion often summarized by the maxim “children are not little adults” can significantly influence drug behavior (Fernandez et al., 2011; Gerhart et al., 2022). For instance, immature phase I/II metabolic enzymes, altered body composition, and underdeveloped renal function can modify the pharmacokinetic trajectories of compounds such as silvestrol in pediatric patients (Schiffmann et al., 2022). Physiologically based pharmacokinetic (PBPK) modeling and population PK studies have been instrumental in predicting pediatric drug exposure and informing dose adjustments (Verscheijden et al., 2020). Therefore, while our computational results offer valuable insights, future work should include pediatric-specific PBPK modeling and empirical PK/safety studies to ensure appropriate dosing and maximize translational relevance in pediatric COVID-19 applications.

Although the findings in this study are promising in terms of inferring the use of Silvestrol as a potential therapeutic agent in treating pediatric COVID-19, some limitations must be mentioned. To begin with, the *in silico* results, which comprise molecular docking and pharmacophore use, do not take into consideration the nuances of *in vivo* conditions; hence, the *in silico* results only provide hints on how things operate. Although computationally characterized as a potent binding agent to the SARS-CoV-2 spike protein, the binding affinity of Silvestrol still needs to be verified experimentally in all the studied forms, including *in vitro* tests and, ultimately, clinical trials to ensure its safety and efficacy in human pediatric cohorts. Moreover, the ADMET profile, as depicted in this paper, is primarily computational in nature. Despite showing potential in preclinical toxicity screenings, Silvestrol requires comprehensive *in vivo* toxicity profiling to determine its safety, particularly when used over the long term. The other limiting factor is the pharmacokinetics of the compound; as good solubility and permeability of Silvestrol have been demonstrated, the bioavailability of this compound and its off-target effects should be studied further.

In the future, prospective research should focus on *in vitro* and *in vivo* confirmation of the antiviral activity and safety of Silvestrol in pediatric models. Preclinical studies on animal models will be crucial in determining the pharmacodynamics, pharmacokinetics, and potential toxicity of the compound when administered to living organisms. Additionally, clinical trials will be required to identify the optimal dosage of Silvestrol and treatment course in children, as well as to assess the long-term implications of Silvestrol on the organism. The prospects of using Silvestrol as a therapeutic agent, however, extend beyond COVID-19, as it may represent a broad-spectrum antiviral agent and could be identified as a possible source of treatment for other viral infections in the pediatric population. Finally, future studies are needed to address such issues and prove its potential as a safe and efficacious antiviral medicine in children.

## Conclusion

Silvestrol proved promising antiviral potential against SARS-CoV-2 through a combination of favorable computational outcomes. While its docking affinity to the receptor-binding domain of the spike protein (−7.5 kcal/mol) indicated a strong initial interaction, this result was further substantiated by molecular dynamics simulations that confirmed stable binding and conformational adaptability, pharmacophore characterization that revealed multiple hydrogen-bonding and hydrophobic interaction sites, and density functional theory (DFT) analysis showing favorable electronic reactivity. Most importantly, the ADMET and toxicity assessments identified Silvestrol as belonging to toxicity class 5 (LD_50_ ≈ 2,300 mg/kg), in contrast to other higher-affinity compounds with more severe predicted toxicities, thereby supporting its suitability for pediatric applications. Taken together, these integrated findings suggest that Silvestrol holds potential as a safe and effective antiviral candidate for pediatric COVID-19, though experimental and clinical validation remain essential.

## Data Availability

The datasets presented in this study can be found in online repositories. The names of the repository/repositories and accession number(s) can be found in the article/Supplementary Material.
