# Supplementary figures and images for: Evaluation of silvestrol as a potential therapeutic agent for pediatric COVID-19: an interpreted computational and phytochemistry approach
Source: Front Pharmacol. 2025 Sep 17;16:1673591. doi: 10.3389/fphar.2025.1673591 (PMC12484239; doi:10.3389/fphar.2025.1673591)

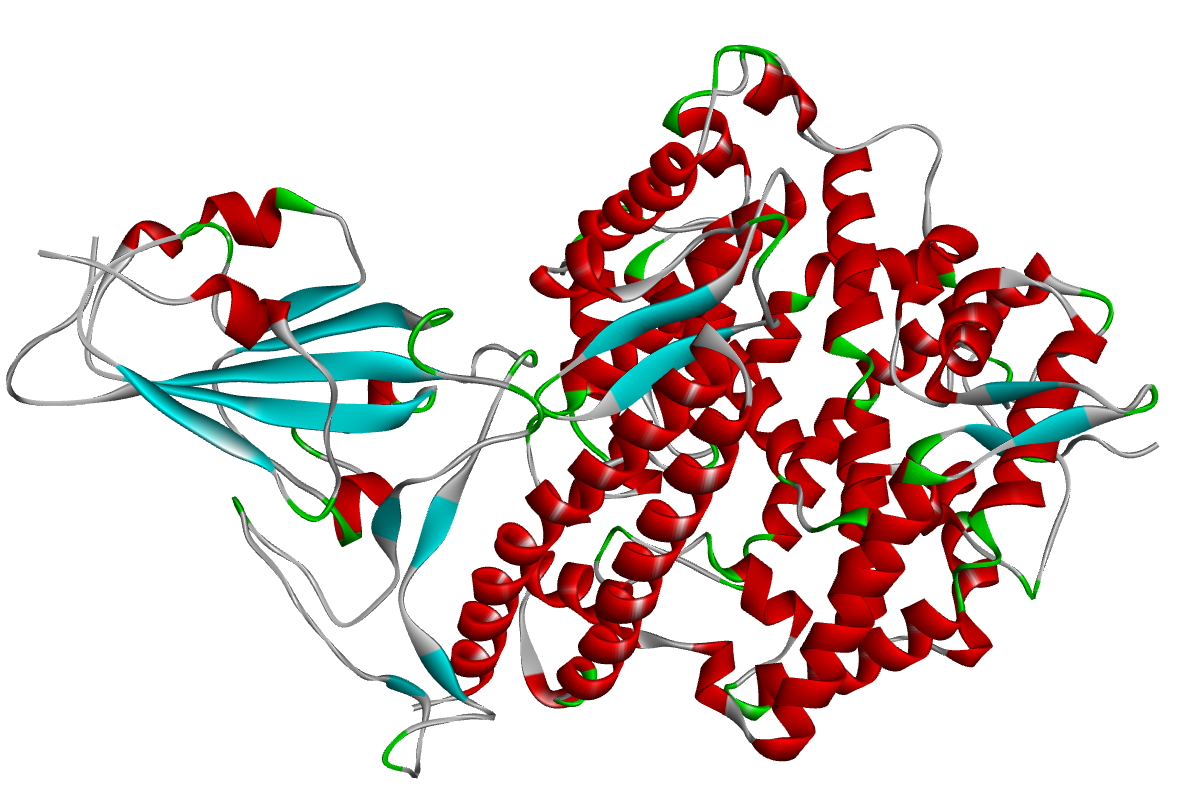

Supplement: Supplementary file 1 [file DataSheet1.zip › Suplementry Data/Receptor/Figure.png]
